# Supplementary material for: Protocol for the process evaluation of the Promoting Activity, Independence and Stability in Early Dementia (PrAISED), following changes required by the COVID-19 pandemic
Source: BMJ Open. 2020 Aug 27;10(8):e039305. doi: 10.1136/bmjopen-2020-039305 (PMC7453764; doi:10.1136/bmjopen-2020-039305)
Supplement: Supplementary data [file bmjopen-2020-039305supp002.pdf]

Appendix 2. Template for analysis of audio-recordings of therapists' remote sessions

Principle	Description	Examples	Rater 1*	Rater 2*
**Intensive**	Physical activity must be performed for at least 150 minutes per week. Participants may require more or less intensive support to achieve this.	Does therapist ask about activity times or amount of activity done over the last week? Do they discuss and agree with participant level of intensity of support required and frequency of next visits? Do they discuss activity plans for the upcoming week?		
**Tailored**	The therapist must work with participant to select and tailor physical exercise / activities that will be of most benefit and interest	Does the therapist make the participant feel they are in control of the activities to be done? For example do they ask whether the participant wants to do the activity? Does the participant seem to enjoy doing it? Is the participant given choices around exercise/activity? Does the therapist make recommendations on activity/exercise based on what the participant has said, or what they have observed the participant do?		
**Challenging**	The tasks must be challenging	Are the tasks challenging enough for participant, but still within their capabilities (i.e. realistically achievable)?		
**Progressive**	The tasks must be progressive	Is the therapist increasing the challenge of the task progressively (even within the same session)? Do they discuss progressing the tasks, now or in the future?		
**Promoting / improving independence**	The tasks must promote or improve independence (ability to complete tasks without dependence on others)	Is the therapist asking the participant to carry out activities independently or working towards them being independent? (e.g. personal, domestic or leisure ADLs, navigating the kitchen, making tea). Do they		

		discuss how the participant could be more independent or set goals for them to do activities independently?		
**Supporting in ADLs or exercise**	The therapist must work with participants to find ways in which the participant can do daily tasks and activities	Does the therapist discuss strategies (e.g. photos, instructions, carer input) with participant to enable them to do their ADLs or exercise? Do they explain how to do them? Do they use a clear language and practical example to support them?		
**Supporting dual-tasking**	The therapist must challenge the participant to complete two exercises at once	Does the therapist ask the participant to do tasks where the mind and the body work at the same time (e.g. walking and counting)? This could be either with the exercise programme or through a functional activity.		
**Accessing the environment**	The therapist must consider ways to maximise physical activity and exercise in the participant's home	Does the therapist ask about, advises on, suggests or gives information on activities that can be done inside the home? Does the therapist discuss full access of the person's property?		
**Embracing positive risk-taking**	Tasks must encourage positive risk-taking and only be discouraged if safety could be compromised	Does the therapist encourage the participant to do tasks where there is a degree of calculated risk? Does the therapist expose the participant to unnecessary risk of harm? Does the therapist discuss positives and negatives of doing more risky activities? Does the therapist use the risk enablement paperwork? Does the therapist consider risk management strategies or contingency plans, when discussing more risky activities?		
**Using Self-Determination Theory principles**	Contact must respond to the human needs for competence (feeling capable of doing the tasks), autonomy (being	Does the therapist give unconditional support and encouragement to boost the participant's confidence? Does the therapist empower the		

	in control of the programme and the tasks)** and relatedness (feeling cared for and connected to the therapist)	participant, by giving them control over the tasks and other aspects of the programme? Is the therapist relatable and working actively to build a human connection with the participant? Does the therapist use the 12 motivational strategies provided by PrAISED team?		
**Assisting in habit formation**	Therapist must assist the participant to develop a habit of being physically active	Does the therapist find ways to integrate the activities into the participant's routine? Do they check that the participant is forming a habit of doing physical activity? Does the therapist use the habit forming strategies provided by PrAISED team?		
**Using tapering to promote self-management**	Therapist must grade the amount of support and supervision provided to participant, to make them more responsible of the activity as time progresses	Does the therapist discuss and agree with participant on the level of intensity of support required to do the tasks and the frequency of next contacts? Is the therapist progressively reducing support (even within the session)? Does the therapist discuss reducing the level of support as programme progresses? Does the therapist use the tapering strategies provided by PrAISED team?		
**Promoting long-term engagement**	The therapist must support the participant to develop intrinsic motivation to ensure that they participants remain active over time	Does the participant seem to enjoy the activity plan? Does it seem that the participants might be able / willing to keep doing the activities over time? Do the therapist work to ensure this, by exploring participant's views?		
**Goal Setting**	The therapist must set goals with the participant that are specific to their interests, functional and active	Does the therapist discuss goal setting with the participant? (sets new goals, review existing goals, adapt/change goals)		

	Does the goal or action plan associated with it lead to the participant doing regular active tasks?		

* Rate as: 1=Visit following principle; 2=visit not following principle; 0=Principle not applicable
