# Supplementary material for: Protocol for the process evaluation of the Promoting Activity, Independence and Stability in Early Dementia (PrAISED), following changes required by the COVID-19 pandemic
Source: BMJ Open. 2020 Aug 27;10(8):e039305. doi: 10.1136/bmjopen-2020-039305 (PMC7453764; doi:10.1136/bmjopen-2020-039305)
Supplement: Author's manuscript [file bmjopen-2020-039305.draft_revisions.pdf]

BMJ Open is committed to open peer review. As part of this commitment we make the peer review history of every article we publish publicly available.

When an article is published we post the peer reviewers' comments and the authors' responses online. We also post the versions of the paper that were used during peer review. These are the versions that the peer review comments apply to.

The versions of the paper that follow are the versions that were submitted during the peer review process. They are not the versions of record or the final published versions. They should not be cited or distributed as the published version of this manuscript.

BMJ Open is an open access journal and the full, final, typeset and author-corrected version of record of the manuscript is available on our site with no access controls, subscription charges or pay-per-view fees (http://bmjopen.bmj.com).

If you have any questions on BMJ Open's open peer review process please email info.bmjopen@bmj.com

BMJ Open

Protocol for the process evaluation of the Promoting Activity, Independence and stability in early Dementia (PrAISED), following changes required by the COVID-19 pandemic

Journal:	*BMJ Open*
Manuscript ID	bmjopen-2020-039305
Article Type:	Protocol
Date Submitted by the Author:	10-Apr-2020
Complete List of Authors:	Di Lorito, Claudio; University of Nottingham,<br>Bosco, Alessandro; University of Nottingham - Jubilee Campus, School of Medicine<br>Goldberg, Sarah; University of Nottingham, Division of Rehabilitation and Ageing, University of Nottingham<br>Nair, Roshan<br>O'Brien, Rebecca<br>Howe, Louise<br>van der Wardt, Veronika; Philipps-Universitat Marburg<br>Pollock, Kristian; University of Nottingham, School of Health Sciences<br>Booth, Vicky<br>Logan, Phillipa; Community Health Sciences<br>Godfrey, Maureen<br>Dunlop, Marianne<br>Horne, Jane<br>Harwood, Rowan; University of Nottingham School of Health Sciences; Nottingham University Hospitals NHS Trust, Health Care of Older People
Keywords:	GERIATRIC MEDICINE, Rehabilitation medicine < INTERNAL MEDICINE, Dementia < NEUROLOGY, REHABILITATION MEDICINE, SPORTS MEDICINE

SCHOLARONE™
Manuscripts

I, the Submitting Author has the right to grant and does grant on behalf of all authors of the Work (as defined in the below author licence), an exclusive licence and/or a non-exclusive licence for contributions from authors who are: i) UK Crown employees; ii) where BMJ has agreed a CC-BY licence shall apply, and/or iii) in accordance with the terms applicable for US Federal Government officers or employees acting as part of their official duties; on a worldwide, perpetual, irrevocable, royalty-free basis to BMJ Publishing Group Ltd ("BMJ") its licensees and where the relevant Journal is co-owned by BMJ to the co-owners of the Journal, to publish the Work in this journal and any other BMJ products and to exploit all rights, as set out in our licence.

The Submitting Author accepts and understands that any supply made under these terms is made by BMJ to the Submitting Author unless you are acting as an employee on behalf of your employer or a postgraduate student of an affiliated institution which is paying any applicable article publishing charge ("APC") for Open Access articles. Where the Submitting Author wishes to make the Work available on an Open Access basis (and intends to pay the relevant APC), the terms of reuse of such Open Access shall be governed by a Creative Commons licence – details of these licences and which Creative Commons licence will apply to this Work are set out in our licence referred to above.

Other than as permitted in any relevant BMJ Author's Self Archiving Policies, I confirm this Work has not been accepted for publication elsewhere, is not being considered for publication elsewhere and does not duplicate material already published. I confirm all authors consent to publication of this Work and authorise the granting of this licence.

For peer review only

Protocol for the process evaluation of the Promoting Activity, Independence and stability in early Dementia (PrAISED), following changes required by the COVID-19 pandemic

Claudio Di Lorito, Alessandro Bosco, Sarah Goldberg, Roshan das Nair, Rebecca O'Brien, Louise Howe, Veronika van der Wardt, Kristian Pollock, Vicky Booth, Pip Logan, Maureen Godfrey, Marianne Dunlop, Jane Horne, Rowan H. Harwood

Abstract

Introduction. The PrAISED Randomised Controlled Trial (RCT) is evaluating a home-based, face-to-face, individually tailored, activity and exercise programme for people living with dementia. Social distancing requirements following the COVID-19 pandemic necessitated rapid changes to intervention delivery.

Methods and analysis. A mixed methods process evaluation will investigate how the changes were implemented and the impact that these have on participants' experience. An *implementation study* will investigate how the therapists delivering the PrAISED intervention were trained and how the intervention was delivered during the pandemic. A *study on the mechanisms of impact and context* will investigate how these changes were experienced by the PrAISED participants, their carers and the therapists delivering the intervention. The study will commence in May 2020.

Ethics and dissemination. The PrAISED RCT and process evaluation have received ethical approval number 18/YH/0059. The ISRCTN Registration Number for PrAISED is 15320670. The PrAISED process evaluation will enable us to understand how distancing and isolation affected participants, their activity and exercise routines, and whether the therapy programme could be continued with remote support. This will be valuable both in explaining trial results, and also contribute to understanding and designing new ways of delivering home-based services and rehabilitation interventions for people with dementia and their carers.

For peer review only

Strengths and limitations of this study

- This process evaluation represents one of the first efforts to document how an ongoing research programme was adapted as a result of the COVID-19 pandemic and how the main stakeholders reacted and adapted to the changes;
- It will contribute knowledge around ways in which people with dementia can be supported to remain physically active and healthy in their homes without face-to-face support;
- It will provide transferable information for researchers to undertake research remotely in such a way that it is ethical, meaningful and practically feasible.

- This process evaluation team is not independent of the main trial team and this may generate confirmation bias of study hypotheses, which will be mitigated by carrying out independent evaluations from different raters.
- In dementia research there is a risk of a lack of ethnic diversity among participants, which will be mitigated, in this study, through purposive sampling.

For peer review only

For peer review only - http://bmjopen.bmj.com/site/about/guidelines.xhtml

Introduction

Dementia is a neurodegenerative condition characterised by a cluster of symptoms, including memory loss and deterioration of motor skills (1-4). More than 50 million people in the world live with dementia (5). Projections estimate that this number will rise to 130 million people in the next 30 years (5). Dementia presents enormous financial burden (6). In the United Kingdom alone, the cost of health and social care for people with the condition is £50 billion, which will grow to £140 billion by 2040 (5). Keeping physically active has benefits for people with dementia on executive functioning, mobility, activities of daily living, independence, and quality of life (7-25), which have been linked to reduced risk of falls, hospital admissions and health and social care costs.

A number of physical activity and exercise intervention programmes have been developed for people with dementia (15;16). Among these is the Promoting Activity, Independence and Stability in Early Dementia (PrAISED) (26), an intervention to promote activity and independence in people with early dementia or mild cognitive impairment, whose clinical and cost-effectiveness is being evaluated in a five-site Randomised controlled Trial (RCT). In brief, 300 participants were randomised to either a control group (brief falls assessment and advice) or an intervention arm (27). Participants in the intervention arm received an individually-tailored programme of up to 50 visits at home over a period of 52 weeks from a multidisciplinary team including physiotherapists (PTs), occupational therapists (OTs) and rehabilitation support workers (RSWs) (27). The PrAISED programme comprises: physical exercises (i.e. progressive strength, balance and dual-task); functional activities (i.e. activities of daily living with an element of physical activity, such as going out for food shopping); promotion of inclusion in community life (e.g. through provision of information on physical exercise group classes); risk enablement (assessing, mitigating and agreeing on risks to be taken or avoided); and environmental assessment (27).

The PrAISED RCT includes a process evaluation (28), which aims to describe and quantify intervention delivery, identify the key elements that make the intervention effective and the variables affecting participants motivation to adhere to the programme and remain physically active in the long-term (i.e. beyond the active intervention period). These variables, which have been recently synthesised in a theoretical model (29,30), include the social opportunities linked to exercise, the therapeutic relationship built up with the therapists delivering the intervention, family or carer support, the availability and inclusion of the person in community (physical) activities, the accessibility of the environment (e.g. availability of parks, public transport) and the notion of independence and autonomy (e.g. how, when, and where to exercise).

In March 2020, many of the elements enabling and supporting participants in the PrAISED programme became impossible to deliver due to the pandemic of COVID-19. Measures to slow the spread of the virus were advised and then mandated by governments (31-33). People over 70 years of age, especially those with pre-existing conditions, were told to self-isolate to shield them from increased risk of illness, complications, hospitalisation and mortality (34, 35).

The negative effects that social isolation may have on the health and well-being of older people are well known (36). In people with dementia, there might be additional effects, such

For peer review only

as a negative impact on functioning, through loss of opportunity to engage with family or in activities outside the home. In order to continue the trial and maintain an element of social contact during this unprecedented time, changes were made to the PrAISED programme intervention training and delivery (Table 1). The main change was that participants would not receive visit from therapists at home, as this would place them at risk of contracting the virus. Instead, therapists would continue to support the participants remotely, by telephone or video, in line with the Chartered Society of Physiotherapists has issued guidance (37). The therapists were provided with written guidance on how to deliver the intervention remotely.

Given the modifications to the intervention, we aim to extend the process evaluation of the PrAISED (28), to investigate the impact of these changes. Specifically, the proposed study will respond to the research questions:

- How does staying at home, with no current possibility of receiving face-to-face support from therapists, affect the uptake and retention of a physical activity and exercise programme in participants with dementia? How does it affect their ability to remain independent and their quality of life? Are there ways in which people with dementia can be better supported to remain physically active and independent in these circumstances?
- How are therapists trained to deliver a physical activity and exercise programme remotely to participants with dementia? How does this training affect their confidence and ability to deliver the intervention? Are there ways in which therapists can better supported to deliver the intervention remotely?

Methods and analysis

Based on the assumption that *'if intervention X (i.e. PrAISED) is delivered, the mediating variable(s) (e.g. staying at home, support from therapists available only remotely) affects the way in which outcome Y (e.g. uptake and retention of a physical activity and exercise) will occur'*, a process evaluation aims to understand how an intervention works (36). It does so by studying the 'implementation of the intervention' (e.g. how the intervention is delivered), the 'mechanisms of impact' (e.g. how participants respond individually to the intervention being delivered) and the 'context' (e.g. the physical and social environment affecting participants' response to the intervention) (38).

This process evaluation will adopt a mixed-methods approach, including quantitative data and data ensuing from qualitative interviews. It will consist of two studies: an implementation study and a study on mechanisms of impact and context (Figure 1).

Patient and Public Involvement

The process evaluation study team includes two patient and public involvement (PPI) contributors (MG and MD), who have been involved in the development of the process evaluation, its protocol (also acting as co-authors). The PPI contributors co-designed with the

For peer review only

main researcher (CDL) the topic guide for the qualitative interviews of participants with dementia and their carers (see details in "study of mechanisms of impact and context – data collection") and will be involved as co-raters in the qualitative analysis of the transcripts of the interviews (see details in "study of mechanisms of impact and context – data analysis") and in disseminating research findings (e.g. through attending conferences, public dissemination events and co-authoring results' papers).

Implementation study

The study on implementation will investigate how the PrAISED training and intervention are delivered, following changes in procedure in response to the COVID-19 pandemic. It will focus on four domains (Table 2):

- Fidelity (i.e. the consistency of training and delivery of PrAISED with the amended protocol);
- Adaptations (i.e. alterations made to training and delivery of PrAISED to achieve better contextual fit);
- Dose (i.e. how much PrAISED training and intervention are delivered);
- Reach (i.e. the number of therapists trained to deliver PrAISED and of participants who receive the intervention).

Participants

The implementation study will include participants with dementia in the intervention group, their carers and therapists, who are involved in the PrAISED main trial at the time of recruitment (May 2020).

Data collection

From the participants with dementia:

- Adherence to intervention as per instructions (Fidelity), investigated through qualitative interviewing;
- Adherence to advised activity levels (Dose), investigated through minutes of PrAISED activity per week as recorded on a self- (or carer-) completed monthly calendar;
- The extent to which participants with dementia come into contact with the intervention (Reach), investigated by totalling the number of participants who completed the programme;
- Alterations that participants made to achieve better contextual fit (Adaptations), investigated through qualitative interviewing.

For peer review only - http://bmjopen.bmj.com/site/about/guidelines.xhtml

From the therapists:

1. Evaluation of the training received following changes made as a result of the pandemic, including:

    - Delivery of training as planned in the original PrAISED protocol (26) (fidelity and dose): hours of training (total and for each site);
    - Attendance of training (fidelity and dose): number and professional role of active therapists attending the training in each site. These data will be gathered through recording attendance to the training sessions.
    - Completion rates of assessment questionnaire (reach): at the end of the PrAISED training sessions, all therapists are asked to complete a questionnaire on the training content. Information on how many attempts are made to pass the questionnaire and the total score for each therapist in the questionnaire will be recorded.
    - Tailoring of training (adaptations): adaptations made to the format of training to respond to the unique circumstance resulting from the COVID-19 pandemic will be recorded.

2. Evaluation of the delivery of adapted intervention as a result of the pandemic, including:

    - Number and length of remote sessions the therapists have with participants (dose and reach): A record of the date, length in minutes, and therapist type (PT, OT and RSW) will be recorded for each contact. The information is collated by the research team each week.
    - Goals set for participants (adaptations): Goals that have been set with the participants are documented by the therapists and collated centrally by the research team.
    - Intervention content (fidelity, adaptations): One intervention session provided remotely by each therapist will be audio-recorded. To ensure safe handling and storing of sensitive data, the session between the therapist and the participant will be recorded remotely by one researcher within the PrAISED team with an encrypted digital audio recorder.

Data analysis

The data from the implementation study will be analysed using IBM SPSS Statistics version 26 (39). Descriptive statistical analysis will be used to measure fidelity, dose and reach.

The audio recordings will be transferred onto an encrypted and password protected university computer server. The content will be assessed independently by two raters against 14 core

For peer review only - http://bmjopen.bmj.com/site/about/guidelines.xhtml

principles set out in the PrAISED therapists' training manual (i.e. *'visit following core principle', 'visit not following core principle', 'Principle not applicable'*). An audio-analysis template will list the core principles, provide operational definitions of each of them, accompanied with practical examples of the application of principle, to facilitate retrieval of content during analysis (Appendix 2).

Prior to independent audio analysis, the two raters will pilot-test the rating procedure using a sample audio recording, to check inter-rater reliability. Scores from the two raters will be compared to determine inter-rater reliability, and if inconsistency arises in scoring, consensus will be reached through discussion between the two raters or through involvement of a third rater.

Study on mechanisms of impact and context

Purposive sampling will be used to gather the full range of perspectives from all the agents included in the intervention and to obtain a sample that is representative of the participants in the RCT (e.g. in relation to the different services involved in PrAISED), following the Medical Research Council (MRC) guidance on process evaluation (38).

Participants

For each research site, we will include:

1. Participants with dementia and their carer, further divided in:

- Intervention arm (i.e. receiving the active intervention);
- Control arm (i.e. receiving treatment as usual);
- Those who have withdrawn from the therapy programme, if they agree to be interviewed.

Given the potential impact on engagement and adherence to the intervention of cohabiting with a carer, as opposed to living independently, participants with different residence status will be selected from the intervention and control groups. Differentiating between subgroups will enable the process evaluation to identify those factors that affect participants' experience of the intervention.

We will not exclude participants who do not have mental capacity to agree to participate or who show fluctuating capacity at the point of the interview, for the following reasons: Firstly, they might still provide precious insight into the mechanisms of the intervention; secondly, their (fluctuating) cognition may have an impact and affects their response toward the intervention; finally, from an ethical standpoint, we aim to give voice to all those whose life is primarily affected by our research. However, we will take into account capacity to give consent (or lack thereof) during the course of the interview, by relying, for example, on different degrees of carer support during the session.

For peer review only - http://bmjopen.bmj.com/site/about/guidelines.xhtml

2. Therapists will be purposively sampled to be involved in the process evaluation, to ensure representation of the different professions (i.e. physiotherapists, occupational therapists and rehabilitation support workers) and of the different research sites.

In line with Guest, Bunce and Johnson (40), we argue that, given the lack of guidance around reaching data saturation, there is a need to adopt appropriate 'tests of adequacy' for sample sizes in qualitative research. Based on the notion of 'conceptual density' (i.e. gathering data until a *sufficient depth* of understanding of the domains under investigation is reached) (41), we will adopt a *Conceptual Depth Scale* developed by Nelson (41) (Table 3), which assigns a score ranging from 1 (low) to 3 (high) to establish whether conceptual density is reached in relation to:

- *'Range'* (e.g. extent of diversity of data sources);
- '*Complexity'* (e.g. extent of networks / links across data);
- '*Subtlety'* (e.g. extent of similarity across data);
- *'Validity'* (e.g. extent to which data are transferable to other settings)

The scoring will be performed by two researchers independently of each other. The scale is used as instrument to check whether consensus is reached among researchers with respect to data saturation, rather than as quantitative assessment to determine a saturation point for data interpretation.

Data collection

The investigation of the mechanisms of impact and context will be based on qualitative interviews with participants. The first interview will be conducted one month following the change of intervention in response to the COVID-19 pandemic (i.e. May 2020). Follow up interviews will be considered, if the measures imposed following the COVID-19 pandemic are still in place, to monitor progress over time.

The interviews will consist of:

- Remote interviews (different options will be offered, including telephone or video calling, depending on participants' preference) with participants with dementia and their carers (as a dyad, so that the carer can provide information, as well as support, if needed). We will use a speaker phone (for everyone to be able to contribute). Prior to the session, the researcher will mail (or email) a copy of the consent form. A verbal consent for both the participant with dementia and the carer will be recorded on tape, before the interview begins.

- Remote interviews with therapists (i.e. occupational therapist, physiotherapist and rehabilitation support worker). Verbal consent will be recorded on tape prior to the interview.

For peer review only - http://bmjopen.bmj.com/site/about/guidelines.xhtml

The topic guide for the qualitative interviews is informed by the *PHYT in dementia* (PHYsical activity behaviour change Theory in dementia), whose development and validation we reported elsewhere (29,30). Through this theoretical framework, we identified potential variables mediating intervention outcomes and developed several prompts to stimulate discussion. Exploration of context will include the impact of isolation, and its effects on exercise, activity and mental well-being.

We developed the topic guides as a collaborative effort between the research team and the PPI contributors, who helped to ensure that the interview prompts are relevant, meaningful and accessible for the participants. Although questions are study-specific, the prompts are broad in scope, to ensure that the participants feel free to express their ideas around unanticipated causal processes and consequences. The participants may also raise additional topics and issues which they feel are particularly relevant in the context of the COVID-19 pandemic, and these will be explored accordingly.

The qualitative interviews are expected to last around 40 minutes, depending on participants' engagement in the process, their cognitive abilities, and logistics.

Data analysis

Data will be analysed through framework analysis (42). This method is ideal in social and health care qualitative research studies with large data sets. Framework analysis will ensure in-depth exploration of data, a transparent audit trail of the process of analysis, and the understanding of data interpretation (e.g. a description of how data link to each other and according to the objective of the study) through visual mapping (42).

Data analysis will follow the steps for good practice in Framework Analysis identified by Gale et al. (42):

1. *Verbatim transcription* of the interviews by a professional transcriber, who will also anonymise data. Large margins and double line spacing in the transcripts will be left to create room for coding and note taking.
2. *Familiarisation with the transcripts* by the main researcher (CDL), who will write down analytical notes on margins.
3. *Coding of a sample of three transcripts* by the main researcher, a second researcher within the research team and one PPI contributor, who will independently underline relevant pieces of text and write coding labels for each, reflecting the constructs included in the topic guide. However, to prevent the omission of important data, if novel constructs are identified from the transcripts, new coding labels will be generated.
4. *Development of a working analytical framework* through team work of the three coders, who will create a set of initial codes through synthesis of individual coding and operational definitions. Two more transcripts will be coded by two coders to check whether the initial working analytical framework is suitable. Eventually, a stable set of codes, clustered into umbrella categories will be identified.

For peer review only - http://bmjopen.bmj.com/site/about/guidelines.xhtml

5. *Use of the working analytical framework* by the main researcher (CDL) to code the whole set of transcripts in NVivo 12 (43). Double coding will be conducted by another researcher.
6. *Charting of data into the framework matrix* by the main researcher on NVivo. The matrix will map out codes (one per column) and participants (one per row). The relevant quotes will be transferred from NVivo onto the matrix.
7. *Interpretation of data* by the main researcher, who will develop themes from the matrix by making connections within and between participants and categories. This will be an iterative process, with regular review from members of the research team.

Ethics and dissemination

The PrAISED trial and process evaluation have received ethical approval number 18/YH/0059. The ISRCTN Registration Number for PrAISED is 15320670.

This protocol, grounded in the MRC framework for process evaluation of complex intervention (38), outlines the rationale, design and methods for the process evaluation of the Promoting Activity, Independence and Stability in Early Dementia and mild cognitive impairment (PrAISED), following the changes made as a result of the restrictions on face-to-face contact during the COVID-19 pandemic.

In only a few months, the COVID-19 pandemic has required dramatic changes to our lifestyles and caused unprecedented operational strain on national health and social care systems. There is a need for scientific evidence to inform research and services in response to the current challenges, as well as preparation for services after the pandemic and potential future events. In these respects, the final process evaluation report, which will be disseminated in scientific journals and to the public (e.g. through public engagement events), will report on the impact that the social distancing measures introduced in PrAISED have had on participants. By comparing the evidence gathered through this study with the original PrAISED process evaluation (28) and the wider literature, findings from this process evaluation will also contribute to the knowledge base around ways in which individuals belonging to the most vulnerable groups in society can be better supported and motivated to remain physically active and healthy in their homes without face-to-face support.

The current evidence shows that face-to-face support from therapists is valued and valuable. Previous studies have found, for example, that the presence of therapists delivering the intervention, who can offer practical guidance on how to perform exercises is linked to participants' intervention uptake (30,44). Home visits from professionals have also been found to facilitate, over time, the creation of a strong therapeutic alliance between the person with dementia, the carer and the therapist, which proves an effective tool for intervention adherence (30). The regular home visits of therapists may also facilitate collaboration with the participant with dementia and the carer in designing a programme tailored to the person's needs and aspirations. Co-production in care has been linked to feelings of empowerment and autonomy in people living with dementia (45).

The literature suggests that home visits may also prove positive for the carers, who have been referred to as "the invisible patients" (46), given the loneliness and social isolation that they

For peer review only - http://bmjopen.bmj.com/site/about/guidelines.xhtml

For peer review only

may experience. Research has established that, as a result of their caring duties, carers may incur into a lack of social contact (47, 48) and reduced quality of life (49). Face-to-face contact with the therapists might present opportunities to combat loneliness by (re)establishing meaningful human connections (44).

There is also mounting evidence that carers may appreciate the practical guidance offered by therapists on how to support the person with dementia to exercise safely and how to deal with challenging situations in the home (e.g. being showed what to do if the person with dementia falls). This type of training may result in decreased feelings of worries, which have been found prominent amongst carers, and linked to restrictive 'gate-keeping' behaviour (50-52). Given that carers represent an invaluable asset and act as enablers of physical activity and exercise in dementia, particularly as the conditions progress (53), the physical presence of the therapist may be crucial to challenge these worries through risk-enabling strategies. The therapists too may benefit from face-to-face interaction with participants. In comparison with typical clinical practice, the length of the intervention (i.e. 12 months in the case of PrAISED) may enable them to know the participants better, and tailor their support to achieve participant-relevant goals (30).

On the other hand, there is evidence in the literature that face-to-face support from therapists can be counterproductive. In light of their long-term relationship with the therapists, the participants may exhibit dependency, potentially resulting in growing worries and anxiety, toward the end of the intervention period (30). Research suggests the salience of attachment theories in the context of dementia, where unwilling separation and disruption of attachment bonds is common (54). Despite the therapists working proactively to prepare participants, upon discontinuation of support the participants might experience feelings of loss and an inability / unwillingness to exercise.

Home visits might also become problematic for therapist-carer rapport. Research has established that carers might hold certain expectations that might be unmet (55). This can set in motion a "cycle of discontent" (55), which can generate poor cooperativeness from carers (30), and deleterious effects on intervention outcomes. From the therapists' perspective, delivering an intervention in the participants' homes can be time consuming. It has been reported in previous process evaluations that adding travelling times on top of the existing workload might have a negative impact on perceived job satisfaction (56). The use of remote support might rectify some of these negative experiences.

This work will also present important implications in theory advancement. Our dissemination plans include a paper further validating the *PHYT in dementia*, the behaviour change theoretical model that our research team previously developed and validated through data from the original PrAISED process evaluation (33,34). Results from this work will contribute further evidence to confirm / challenge the validity of the model in explaining motivation to be physically active, in the context of social distancing. Finally, based on findings from this process evaluation, we aim to develop a methodological paper outlining strategies that can be used to involve research participants remotely in an ethical, meaningful and practically feasible way. This model can be refined through input from research teams conducting rehabilitation studies in similar circumstances, such as the FinCH study (57), to derive a research platform that can be shared to inform / guide good practice in future research.

For peer review only - http://bmjopen.bmj.com/site/about/guidelines.xhtml

In conclusion, this process evaluation represents one of the first efforts to document how an ongoing research programme was adapted as a result of the COVID-19 pandemic. This study will support the critical reflection by the PrAISED team on positive and negative aspects of these adaptations. It will also provide transferable information to develop strategies to effectively deliver rehabilitation remotely, in the presence of extraordinary circumstances (e.g. social distancing and staying at home).

Funding statement: This protocol presents independent research funded by the United Kingdom National Institute for Health Research (NIHR) under its Programme Grants for Applied Research funding scheme (RP-PG-0614-20007). The views expressed are those of the authors and not necessarily those of the National Health Service, the NIHR or the Department of Health and Social Care.

Conflict of interest: None to declare

Authors' contributions: All the co-authors have equally contributed to the development of the protocol of this process evaluation and have seen and approved the final version.

Words: 3938

For peer review only

For peer review only - http://bmjopen.bmj.com/site/about/guidelines.xhtml

Reference

(1). Martyr, A. and L. Clare, Executive function and activities of daily living in Alzheimer's disease: a correlational meta-analysis. Dementia and geriatric cognitive disorders, 2012. 33(2-3): p. 189-203.

(2). Giebel, C.M., et al., Deterioration of basic activities of daily living and their impact on quality of life across different cognitive stages of dementia: a European study. International Psychogeriatrics, 2014. 26(8): p. 1283-1293.

(3). Giebel, C.M., C. Sutcliffe, and D. Challis, Activities of daily living and quality of life across different stages of dementia: a UK study. Aging & Mental Health, 2015. 19(1): p. 63-71.

(4). Alzheimer's Research UK (2019). About dementia. Available online: https://www.alzheimersresearchuk.org/about-dementia/?gclid=Cj0KCQjw3JXtBRC8ARIsAEBHg4myuYeahFMLCcSGLr-flQxznmdB-dObW2gXc5MUN9o_dfNw5wwI5EwaAvH6EALw_wcB (accessed on 26 March 2020).

(5). Alzheimer's Society (2020). Facts for the Media. Available online: https://www.alzheimers.org.uk/about-us/news-and-media/facts-media (accessed on 26 March 2020).

(6). Lewis F, Karlsberg Schaffer S, Sussex J, O'Neill P, Cockcroft L. The trajectory of dementia in the UK-making a difference. Office of Health Economics Consulting Reports. 2014 Jun 1.

(7). Forbes D, Forbes SC, Blake CM, Thiessen EJ, Forbes S. Exercise programs for people with dementia. Cochrane Database of Systematic Reviews. 2015(4). doi:10.1002/14651858.CD006489.pub4.

(8). Potter R, Ellard D, Rees K, Thorogood M. A systematic review of the effects of physical activity on physical functioning, quality of life and depression in older people with dementia. International journal of geriatric psychiatry. 2011 Oct;26(10):1000-11.

(9). Blankevoort CG, Van Heuvelen MJ, Boersma F, Luning H, De Jong J, Scherder EJ. Review of effects of physical activity on strength, balance, mobility and ADL performance in elderly subjects with dementia. Dementia and geriatric cognitive disorders. 2010;30(5):392-402.

(10). Heyn P, Abreu BC, Ottenbacher KJ. The effects of exercise training on elderly persons with cognitive impairment and dementia: a meta-analysis. Archives of physical medicine and rehabilitation. 2004 Oct 1;85(10):1694-704.

(11). Hauer K, Schwenk M, Zieschang T, Essig M, Becker C, Oster P. Physical training improves motor performance in people with dementia: a randomized controlled trial. Journal of the American Geriatrics Society. 2012 Jan;60(1):8-15.

(12). Hoffmann K, Sobol NA, Frederiksen KS, Beyer N, Vogel A, Vestergaard K, Brændgaard H, Gottrup H, Lolk A, Wermuth L, Jacobsen S. Moderate-to-high intensity

For peer review only - http://bmjopen.bmj.com/site/about/guidelines.xhtml

physical exercise in patients with Alzheimer's disease: a randomized controlled trial. Journal of Alzheimer's Disease. 2016 Jan 1;50(2):443-53.

(13). Lamb SE, Sheehan B, Atherton N, Nichols V, Collins H, Mistry D, Dosanjh S, Slowther AM, Khan I, Petrou S, Lall R. Dementia And Physical Activity (DAPA) trial of moderate to high intensity exercise training for people with dementia: randomised controlled trial. bmj. 2018 May 16;361:k1675.

(14). Lowery, D., Cerga-Pashoja, A., Iliffe, S., Thuné-Boyle, I., Griffin, M., Lee, J., Bailey, A., Bhattacharya, R. and Warner, J., 2014. The effect of exercise on behavioural and psychological symptoms of dementia: the EVIDEM-E randomised controlled clinical trial. *International journal of geriatric psychiatry*, *29*(8), pp.819-827.

(15). Pitkälä KH, Pöysti MM, Laakkonen ML, Tilvis RS, Savikko N, Kautiainen H, Strandberg TE. Effects of the Finnish Alzheimer disease exercise trial (FINALEX): a randomized controlled trial. JAMA internal medicine. 2013 May 27;173(10):894-901.

(16). Prick AE, De Lange J, Scherder E, Twisk J, Pot AM. The effects of a multicomponent dyadic intervention with physical exercise on the cognitive functioning of people with dementia: A randomized controlled trial. Journal of aging and physical activity. 2017 Oct 1;25(4):539-52.

(17). Rolland Y, Pillard F, Klapouszczak A, Reynish E, Thomas D, Andrieu S, Rivière D, Vellas B. Exercise program for nursing home residents with Alzheimer's disease: a 1-year randomized, controlled trial. Journal of the American Geriatrics Society. 2007 Feb;55(2):158-65.

(18). Schwenk M, Zieschang T, Oster P, Hauer K. Dual-task performances can be improved in patients with dementia: a randomized controlled trial. Neurology. 2010 Jun 15;74(24):1961-8.

(19) Schwenk M, Zieschang T, Englert S, Grewal G, Najafi B, Hauer K. Improvements in gait characteristics after intensive resistance and functional training in people with dementia: a randomised controlled trial. BMC geriatrics. 2014 Dec;14(1):73.

(20). Suzuki T, Shimada H, Makizako H, Doi T, Yoshida D, Tsutsumimoto K, Anan Y, Uemura K, Lee S, Park H. Effects of multicomponent exercise on cognitive function in older adults with amnestic mild cognitive impairment: a randomized controlled trial. BMC neurology. 2012 Dec 1;12(1):128.

(21). Telenius EW, Engedal K, Bergland A. Effect of a high-intensity exercise program on physical function and mental health in nursing home residents with dementia: an assessor blinded randomized controlled trial. PloS one. 2015;10(5).

(22). Forbes D, Forbes SC, Blake CM, Thiessen EJ, Forbes S. Exercise programs for people with dementia. Cochrane Database of Systematic Reviews. 2015(4).

(23). Potter R, Ellard D, Rees K, Thorogood M. A systematic review of the effects of physical activity on physical functioning, quality of life and depression in older people with dementia. International journal of geriatric psychiatry. 2011 Oct;26(10):1000-11.

For peer review only

(24). Blankevoort CG, Van Heuvelen MJ, Boersma F, Luning H, De Jong J, Scherder EJ. Review of effects of physical activity on strength, balance, mobility and ADL performance in elderly subjects with dementia. Dementia and geriatric cognitive disorders. 2010;30(5):392-402.

(25). Heyn P, Abreu BC, Ottenbacher KJ. The effects of exercise training on elderly persons with cognitive impairment and dementia: a meta-analysis. Archives of physical medicine and rehabilitation. 2004 Oct 1;85(10):1694-704.

(26). Booth V, Harwood RH, Hood-Moore V, Bramley T, Hancox JE, Robertson K, Hall J, Van Der Wardt V, Logan P. Promoting Activity, Independence and Stability in Early Dementia and Mild Cognitive Impairment (PrAISED): Development of an Intervention for People with Mild Cognitive Impairment and Dementia. Clinical Rehabilitation 2018; 32: 855-864. doi.org/10.1177/0269215518758149

(27). Bajwa RK, Goldberg SE, Van der Wardt V, Burgon C, Di Lorito C, Godfrey M, Dunlop M, Logan P, Masud T, Gladman J, Smith H. A randomised controlled trial of an exercise intervention promoting activity, independence and stability in older adults with mild cognitive impairment and early dementia (PrAISED)-A Protocol. Trials. 2019 Dec;20(1):1-1.

(28). Di Lorito C, Pollock K, Harwood R, das Nair R, Logan P, Goldberg S, Booth V, Godfrey M, Dunlop M, Van der Wardt V. Protocol for the process evaluation of the promoting activity, independence and stability in early dementia and mild cognitive impairment (PrAISED 2) randomised controlled trial. Maturitas. 2019 Apr 1;122:8-21.

(29). Di Lorito C, Pollock K, Harwood R, das Nair R, Logan P, Goldberg S, Booth V, Vedhara K, Van Der Wardt V. A scoping review of behaviour change theories in adults without dementia to adapt and develop the 'PHYT in dementia', a model promoting physical activity in people with dementia. Maturitas. 2019 Mar 1;121:101-13.

(30). Di Lorito C, Bosco A, Pollock K, H Harwood R, Das Nair R, Logan P, Goldberg S, Booth V, Vedhara K, Godfrey M, Dunlop M. External Validation of the 'PHYT in Dementia', a Theoretical Model Promoting Physical Activity in People with Dementia. International Journal of Environmental Research and Public Health. 2020 Jan;17(5):1544.

(31). World Health Organization (2020). WHO Director-General's opening remarks at the media briefing on COVID-19 - 11 March 2020. Retrieved from: https://www.who.int/dg/speeches/detail/who-director-general-s-opening-remarks-at-the-media-briefing-on-covid-19---11-march-2020 (accessed: 02 April 2020).

(32). Ferguson NM, Laydon D, Nedjati-Gilani G, Imai N, Ainslie K, Baguelin M, Bhatia S, Boonyasiri A, Cucunubá Z, Cuomo-Dannenburg G, Dighe A. Impact of Non-Pharmaceutical Interventions (NPIs) to Reduce COVID-19 Mortality and Healthcare Demand (16 March 2020). URL https://doi. org/10.25561/77482.

(33). Sun P, Lu X, Xu C, Sun W, Pan B. Understanding of COVID-19 based on current evidence. Journal of medical virology. 2020 Feb 25.

(34). Public Health England (2020). Guidance on social distancing for everyone in the UK. Available at: https://www.gov.uk/government/publications/covid-19-guidance-on-social-

For peer review only - http://bmjopen.bmj.com/site/about/guidelines.xhtml

distancing-and-for-vulnerable-people/guidance-on-social-distancing-for-everyone-in-the-uk-and-protecting-older-people-and-vulnerable-adults (Accessed: 24 March 2020)

(35). Zu ZY, Jiang MD, Xu PP, Chen W, Ni QQ, Lu GM, Zhang LJ. Coronavirus disease 2019 (COVID-19): a perspective from China. Radiology. 2020 Feb 21:200490.

(36). Hawton A, Green C, Dickens AP, Richards SH, Taylor RS, Edwards R, Greaves CJ, Campbell JL. The impact of social isolation on the health status and health-related quality of life of older people. Quality of Life Research. 2011 Feb 1;20(1):57-67.

(37) The Chartered Society of Physiotherapy. COVID-19: guide for rapid implementation of remote consultations. Retrieved from: https://www.csp.org.uk/publications/covid-19-guide-rapid-implementation-remote-consultations Accessed on 09 April 2020.

(38). Moore GF, Audrey S, Barker M, Bond L, Bonell C, Hardeman W, Moore L, O'Cathain A, Tinati T, Wight D, Baird J. Process evaluation of complex interventions: Medical Research Council guidance. bmj. 2015 Mar 19;350.

(39). IBM Corp. Released 2017. IBM SPSS Statistics for Windows, Version 25.0. Armonk, NY: IBM Corp.

(40). Guest G, Bunce A, Johnson L. How many interviews are enough? An experiment with data saturation and variability. Field methods. 2006 Feb;18(1):59-82.

(41). Nelson J. Using conceptual depth criteria: addressing the challenge of reaching saturation in qualitative research. Qualitative research. 2017 Oct;17(5):554-70.

(42). Gale NK, Heath G, Cameron E, Rashid S, Redwood S. Using the framework method for the analysis of qualitative data in multi-disciplinary health research. BMC medical research methodology. 2013 Dec;13(1):117.

(43). NVivo qualitative data analysis Software (2018). Melbourne, Australia: QSR International Pty Ltd.

(44). Prick AE, de Lange J, van't Leven N, Pot AM. Process evaluation of a multicomponent dyadic intervention study with exercise and support for people with dementia and their family caregivers. Trials. 2014 Dec 1;15(1):401.

(45). Bosco A, Schneider J, Coleston-Shields DM, Orrell M. Dementia care model: Promoting personhood through co-production. Archives of gerontology and geriatrics. 2019 Mar 1;81:59-73.

(46). Brodaty H, Donkin M. Family caregivers of people with dementia. Dialogues in clinical neuroscience. 2009 Jun;11(2):217.

(47). Brodaty H, Hadzi-Pavlovic D. Psychosocial effects on carers of living with persons with dementia. Australian & New Zealand Journal of Psychiatry. 1990 Sep;24(3):351-61.

(48). Graham C, Ballard C, Sham P. Carers' knowledge of dementia, their coping strategies and morbidity. International journal of geriatric psychiatry. 1997 Sep;12(9):931-6.

(49). Gaugler JE, Kane RL, Kane RA, Newcomer R. Unmet care needs and key outcomes in dementia. Journal of the American Geriatrics Society. 2005 Dec;53(12):2098-105.

For peer review only

(50). Mcintyre A, Reynolds F. There's no apprenticeship for Alzheimer's: the caring relationship when an older person experiencing dementia falls. Ageing & Society. 2012 Jul;32(5):873-96..

(51). Faes MC, Reelick MF, Joosten-Weyn Banningh LW, Gier MD, Esselink RA, Olde Rikkert MG. Qualitative study on the impact of falling in frail older persons and family caregivers: foundations for an intervention to prevent falls. Aging & mental health. 2010 Sep 1;14(7):834-42.

(52). Kuzuya M, Masuda Y, Hirakawa Y, Iwata M, Enoki H, Hasegawa J, Izawa S, Iguchi A. Falls of the elderly are associated with burden of caregivers in the community. International Journal of Geriatric Psychiatry: A journal of the psychiatry of late life and allied sciences. 2006 Aug;21(8):740-5.

(53). Peach T, Pollock K, van der Wardt V, das Nair R, Logan P, Harwood RH. Attitudes of older people with mild dementia and mild cognitive impairment and their relatives about falls risk and prevention: A qualitative study. PLoS One. 2017;12(5).

(54). Browne CJ, Shlosberg E. Attachment theory, ageing and dementia: A review of the literature. Aging and Mental Health. 2006 Mar 1;10(2):134-42.

(55). Jurgens FJ, Clissett P, Gladman JR, Harwood RH. Why are family carers of people with dementia dissatisfied with general hospital care? A qualitative study. BMC geriatrics. 2012 Dec 1;12(1):57.

(56). Bosco A, Paulauskaite L, Hall I, Crabtree J, Soni S, Biswas A, Cooper V, Poppe M, King M, Strydom A, Crawford MJ. Process evaluation of a randomised controlled trial of PBS-based staff training for challenging behaviour in adults with intellectual disability. PloS one. 2019;14(8).

(57). Logan P, McCartney K, Armstrong S, Clarke A, Conroy S, Darby J….& Horne J. Evaluation of the Guide to Action Care Home fall prevention programme in care homes for older people: protocol for a multi-centre, single blinded, cluster randomised controlled trial (FinCH). East Midlands Research into Ageing Network (EMRAN) Discussion Paper Series Issue 25 Feb 2019. ISSN 2059-3341.

For peer review only

For peer review only - http://bmjopen.bmj.com/site/about/guidelines.xhtml

Figure 1. Method of Process Evaluation

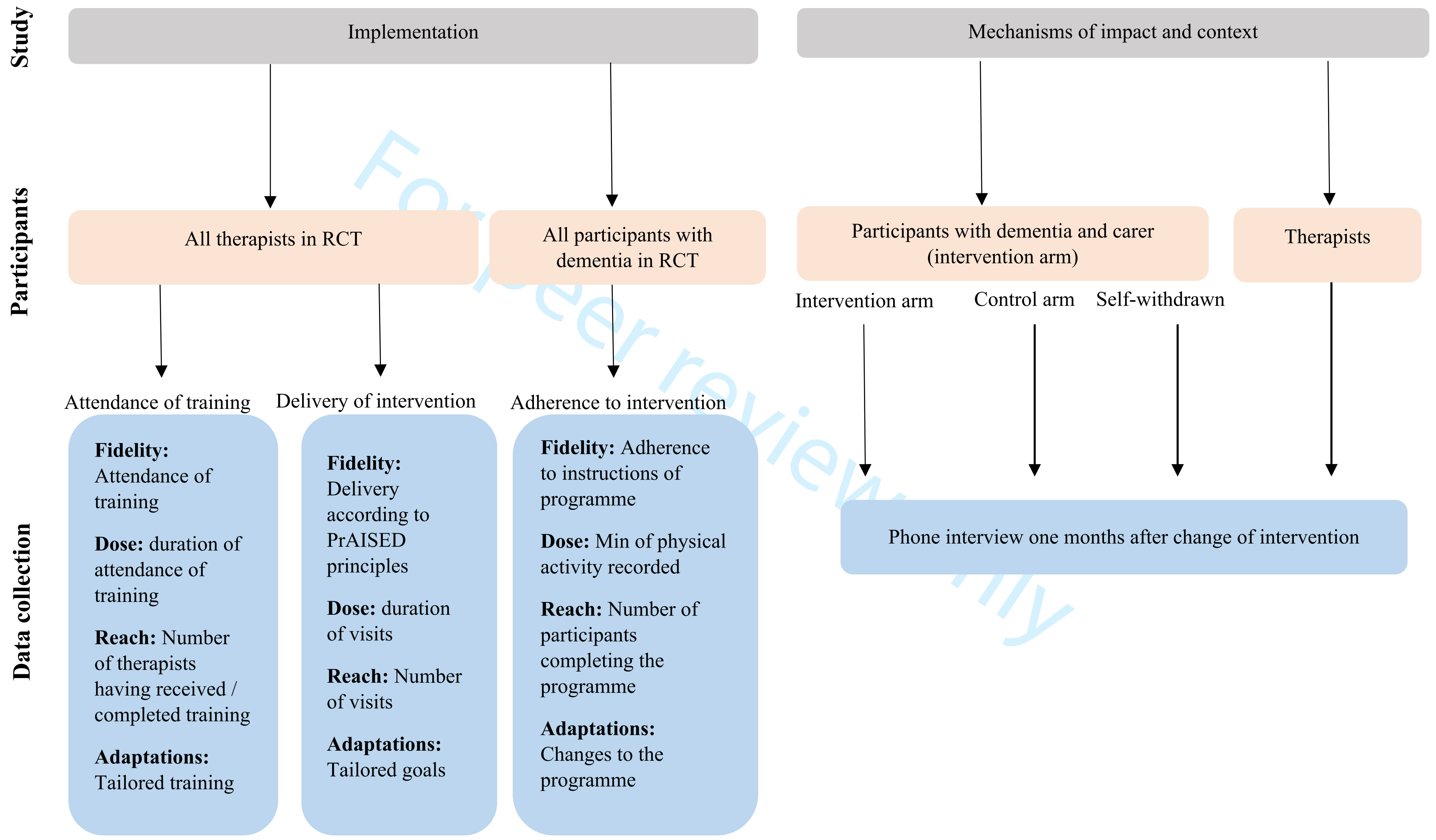

For peer review only - http://bmjopen.bmj.com/site/about/guidelines.xhtml

Table 1. Main changes made to the PrAISED intervention, compared to the original version (31)

Delivery of training	Delivery of intervention	Provision of support to the therapists
No changes, as all therapists delivering PrAISED were recruited and trained before the amendment to PrAISED, following the COVID-19 pandemic	The therapists were provided with written guidance on how to deliver the intervention (Appendix 1)	Increased access to:<br>• Monthly teleconferences across all sites;<br>• Teleconferences at individual sites;<br>• Provision of a regularly updated list of resources;<br>• Provision of informal support through email and phone;<br>• Provision of information and support tailored to the situation and change in practice

For peer review only - http://bmjopen.bmj.com/site/about/guidelines.xhtml

Table 2. Implementation study

	Training		*Intervention*	
	Delivery (PrAISED 2 team)	Attendance (Therapists)	Delivery (Therapists)	Adherence (Participants)
Fidelity	Delivery of training*	Attendance of training*	Delivery of intervention against PrAISED 2 principles (through audio content)	Adherence to intervention as per instructions (through interview)
Dose	Days / hours of training per site*	Days / hours of attendance per therapist*	Frequency and length of contact sessions with participant*	Minutes per week recorded on calendar*
Reach	Number of sites and therapists receiving training*	Number of therapists attending training and number of therapists completing training tasks*	Number of contact sessions with participant*	Number of participants who completed the programme*
Adaptations	Adaptations made when providing training*	Adaptations made to attend training*	Adaptations made to deliver the sessions (through interview)	Adaptations that participants made to physical activity and exercise (through interview)

* Data gathered during main Trial

Table 3. Conceptual Depth Scale (Nelson, 2016)

For peer review only - http://bmjopen.bmj.com/site/about/guidelines.xhtml

**Criteria (with sources of evidence)**	**Low (1)**	**Medium (2)**	**High (3)**
Range (e.g. frequency and variety of codes; multiplicity of data sources)	Few examples to sup-port concepts. Only a single data-type	→	Abundant examples to sup-port concepts. Multiple data-types
Complexity (e.g. coding trees; positional maps; matrices)	Descriptive codes; simple or basic connections between codes; low level analysis	→	Sophisticated networks; abstract conceptual categories which synthesise a range of codes and concepts
Subtlety (e.g. memos; social worlds diagrams)	Conceptual language is regarded as unproblematic and one dimensional	→	Conceptual language is understood as rich, ambiguous and multi-dimensional
Resonance (literature)	Weak resonance; emerging theory is re-mote from existing literature and theoretical frameworks	→	Strong resonance; emerging theory makes sense along-side existing literature; there are correlations with other theoretical frameworks, albeit with variations and novel-ties
Validity (e.g. applicability test)	Low level theorising and inward facing; the findings have limited application to the re-search participants or those familiar with similar contexts.	→	Abstract level theorising and outward facing; the findings make sense to those in the social context of the re-search, or ones broadly similar.

For peer review only

Appendix 1. PrAISED Protocol Supplement, COVID-19 specific, v1 26/03/2020

In response to the COVID19 pandemic, on 16th March 2020, the Government strongly advised that people over 70 years old socially distance themselves to reduce their risk of contracting COVID19. In response to this, the NIHR stated that their funded trials should stop all non-essential face to face contact. This poses two problems for the PrAISED2 trial – how we deliver therapy and how we follow up participants.

Our risk assessment of this situation concludes that face to face visits are not essential clinical care and pose a risk to the safety of our participants. However, our participants are at risk of deconditioning, and to do no exercise during a prolonged period of isolation would be detrimental. We have a duty of care to participants in the trial, and we have therefore developed a protocol for delivering the PrAISED2 intervention via telephone or video coaching. This will be delivered by therapists and rehabilitation support workers at study sites. If PrAISED therapists are redeployed into NHS clinical care due to COVID19, any PrAISED trained therapists across sites could deliver the intervention remotely.

To accommodate restrictions on face to face meetings with participants, we will complete follow up measures remotely. We will telephone the participants, and then with their permission questionnaires will be sent out and then completed by either the researcher telephoning the participant, or by the participant completing the questionnaire and returning by post. We have additionally introduced an interim follow up, which will be completed on all participants who had been in the trial between 3 and 10.5 months on 20th March 2020 (the date the new procedure was approved by HRA). This will be completed as soon as possible, to try and capture any benefits of the intervention before social isolation and potentially contracting COVID19 affects outcomes. We will seek verbal consent from the carer and patient participant to complete this questionnaire. We do not intend to do an interim follow up on participants who have been in the trial for less than 3 months, as it is unlikely that they will have had any benefits from the PrAISED2 intervention. The interim follow up questionnaire will be completed remotely and is based on the current follow up questionnaire, but with the following changes:

i) We have removed all measures that cannot be done remotely (BERG Balance, TUG, Frailty, Blood Pressure, CANTAB, MoCA, verbal fluency).

ii) We have included in the informant questionnaire three questions related to COVID19. We will ask carer participants if they believe the patient participant has had COVID19, if the patient participant has been social distancing and if they have isolated themselves, and for how many days.

iii) We have removed the BFI personality questionnaire, and changed the CSRI format to make it easier for carers to follow. We have also changed the medical history questions to 'ever' rather than 'last 12 months/since baseline' to avoid problems of participants remembering when they were diagnosed with conditions.

Any participants who are currently within six weeks of the 12 month follow up will be followed up immediately using the interim remote follow up questionnaire.

The government also requested that everyone socially distances themselves and wherever possible works from home. In response to this, the University of Nottingham has instructed

For peer review only - http://bmjopen.bmj.com/site/about/guidelines.xhtml

all but essential staff to work from home and is shutting down large parts of the University. This makes access to post rooms more difficult and we are concerned that participants may report adverse events via calendars which we will not receive in a timely way. We have amended the calendar information to ask participants to ring the research team if they have had an adverse event.

For peer review only

For peer review only - http://bmjopen.bmj.com/site/about/guidelines.xhtml

Appendix 1. Guidance distributed among the PrAISED therapists on the changes made to the intervention

Plan for PrAISED2 Intervention in response to COVID19 restrictions (18.03.2020)

Immediate plan

The NIHR have stated that their funded studies should stop all non-essential face to face contact. The PrAISED intervention is not considered essential care and therefore we must stop all face to face contact with our participants.

However, because we have a duty of care to our patients considering many of them will be following the governments advice to reduce all social contact, we have devised a contingency plan to continue with the PrAISED intervention.

Intervention Group Participants

Therapy teams should contact all participants currently in the trial, or their carers if more appropriate, to explain the change in practice as below.

On-going Intervention Group Participants

Visits to participants should be replaced with **telephone coaching** as per their normal schedule, in terms of frequency. For example, if you are seeing someone weekly, this should be continued until they reach the time to reduce to fortnightly. This is the example frequency schedule set out in the intervention manual, however, continue to adapt this as appropriate in the same way you have been doing.

- Month 1-2: bi-weekly
- Month 3-6: weekly
- Month 6-9: fortnightly
- Month 9-12: monthly

The length of the phone call may be much shorter depending on what is discussed.

The content of the phone call should be guided by the telephone coaching instructions below.

Some participants won't be suitable for telephone calls. If the participant is unable to engage with telephone coaching the carer should be contacted to determine if they may be able to use the telephone coaching to support the participant. If the telephone coaching is of no benefit to either the participant or the carer, then a courtesy telephone call should be given each month to keep in touch with the carer or participant as appropriate.

Final sessions should be carried out via the telephone as appropriate; these should be followed up with an end of therapy letter and any follow up material being provided using the post or email if appropriate.

For peer review only - http://bmjopen.bmj.com/site/about/guidelines.xhtml

New Intervention Group Participants

Intervention group participants seen by the research team but not yet seen by therapy team, or who are in the assessment phase of the intervention, should be informed that they are not going to receive the PrAISED intervention until the current restrictions are lifted.

Control Group Participants

If you have completed the first control visit you can carry out up to two follow up visits by telephone as per the guidance below. If the first control visit has not yet been completed, please inform the participant that they are not going to receive the PrAISED intervention until the current restrictions are lifted.

Therapy Visit Log

Continue to complete the therapy visit log, via the hyperlink, for all telephone calls. Please put telephone coaching in the comments box.

Medium-Term Plan

It is expected that PrAISED therapy staff at each site will deliver the immediate plan outlined above.

However, as the situation changes a medium-term plan (outlined below) may come into action.

If sites cannot deliver the telephone coaching sessions due to therapy staffing difficulties, the university staff may have capacity to be able to support. The PI from each site must contact the University as soon as possible if this happens. For university staff to be able to do the telephone coaching sessions effectively, we will need to know:

- the participant's details (e.g., contact telephone number for them and the carer/informant)
- a synopsis of the previous intervention session and what they are currently working on

As each site is using different participant documentation systems, the PIs should liaise with Sarah Goldberg or Rebecca O'Brien, to form a contingency plan on how this will happen and how information is to be transferred and stored.

Telephone Coaching Instructions

Before making the telephone call make sure you have looked at NHS England current advice for the client group you are dealing with, as this is likely to change on a regular basis (https://www.nhs.uk/conditions/coronavirus-covid-19/). Participants may have concerns about their current situation that need answering before the participant will engage in coaching.

For peer review only - http://bmjopen.bmj.com/site/about/guidelines.xhtml

- Explain who you are and why you're calling.
- Ask how they are and discuss any immediate concerns (they may need signposting as appropriate).
- Review their current activity and exercise plan.
- Review what they are currently doing during their day.
- Be aware that for many participants all their activities may have stopped.
- Form a plan of what they can do within the **current** restrictions. For example, currently people are advised it is ok to walk outside as long as they stay 2m away from other people.
- Help them to make a daily plan of activities. For example, doing exercises more frequently, or if they are no longer walking outside can they walk in the garden or up and down the stairs to get some cardiovascular exercise.
- Advise against sitting for long periods of time. For example, use a timer to remind yourself to get up or get up during advert breaks in television programmes.
- If the person is able to and wants to, they could put you on speaker phone while you go through their exercise programme with them. Only do this if they have the capacity to do this with their telephone. This could also be done with their carer or family member or named informant.
- Be aware people may be feeling quite worried and/or low in mood. You may need to discuss the benefits of, and encourage them to continue to carry out daily activities or routines, such as getting dressed, or taking meals on time.
- Participants may raise safeguarding issues such as identifying they are low on medication and there is no one to help them with this. This will need to addressed using the usual safeguarding procedures.
- If participants are complaining of COVID 19 symptoms they should be encouraged to follow the current advice from NHS direct or to phone 111.

It is expected these telephone coaching guidelines will evolve as PrAISED therapists start conducting these sessions. Guidance can come from outside sources, e.g., RCOT have recently shared this online https://www.rcot.co.uk/staying-well-when-social-distancing. It is important that we share good practice and suggestions and will discuss these guidelines during our PrAISED Therapist Teleconferences.

For peer review only - http://bmjopen.bmj.com/site/about/guidelines.xhtml

Appendix 2. Template for analysis of audio-recordings of therapists' remote sessions

Principle	Description	Examples	Rater 1*	Rater 2*
**Intensive**	Physical activity must be performed for at least 150 minutes per week. Participants may require more or less intensive support to achieve this.	Does therapist ask about activity times or amount of activity done over the last week? Do they discuss and agree with participant level of intensity of support required and frequency of next visits? Do they discuss activity plans for the upcoming week?		
**Tailored**	The therapist must work with participant to select and tailor physical exercise / activities that will be of most benefit and interest	Does the therapist make the participant feel they are in control of the activities to be done? For example do they ask whether the participant wants to do the activity? Does the participant seem to enjoy doing it? Is the participant given choices around exercise/activity? Does the therapist make recommendations on activity/exercise based on what the participant has said, or what they have observed the participant do?		
**Challenging**	The tasks must be challenging	Are the tasks challenging enough for participant, but still within their capabilities (i.e. realistically achievable)?		
**Progressive**	The tasks must be progressive	Is the therapist increasing the challenge of the task progressively (even within the same session)? Do they discuss progressing the tasks, now or in the future?		
**Promoting / improving independence**	The tasks must promote or improve independence (ability to complete tasks without dependence on others)	Is the therapist asking the participant to carry out activities independently or working towards them being independent? (e.g. personal, domestic or leisure ADLs, navigating the kitchen, making tea). Do they		

For peer review only

		discuss how the participant could be more independent or set goals for them to do activities independently?		
**Supporting in ADLs or exercise**	The therapist must work with participants to find ways in which the participant can do daily tasks and activities	Does the therapist discuss strategies (e.g. photos, instructions, carer input) with participant to enable them to do their ADLs or exercise? Do they explain how to do them? Do they use a clear language and practical example to support them?		
**Supporting dual-tasking**	The therapist must challenge the participant to complete two exercises at once	Does the therapist ask the participant to do tasks where the mind and the body work at the same time (e.g. walking and counting)? This could be either with the exercise programme or through a functional activity.		
**Accessing the environment**	The therapist must consider ways to maximise physical activity and exercise in the participant's home	Does the therapist ask about, advises on, suggests or gives information on activities that can be done inside the home? Does the therapist discuss full access of the person's property?		
**Embracing positive risk-taking**	Tasks must encourage positive risk-taking and only be discouraged if safety could be compromised	Does the therapist encourage the participant to do tasks where there is a degree of calculated risk? Does the therapist expose the participant to unnecessary risk of harm?<br>Does the therapist discuss positives and negatives of doing more risky activities?<br>Does the therapist use the risk enablement paperwork? Does the therapist consider risk management strategies or contingency plans, when discussing more risky activities?		
**Using Self-Determination Theory principles**	Contact must respond to the human needs for competence (feeling capable of doing the tasks), autonomy (being	Does the therapist give unconditional support and encouragement to boost the participant's confidence? Does the therapist empower the		

For peer review only

	in control of the programme and the tasks)** and relatedness (feeling cared for and connected to the therapist)	participant, by giving them control over the tasks and other aspects of the programme? Is the therapist relatable and working actively to build a human connection with the participant? Does the therapist use the 12 motivational strategies provided by PrAISED team?		
**Assisting in habit formation**	Therapist must assist the participant to develop a habit of being physically active	Does the therapist find ways to integrate the activities into the participant's routine? Do they check that the participant is forming a habit of doing physical activity? Does the therapist use the habit forming strategies provided by PrAISED team?		
**Using tapering to promote self-management**	Therapist must grade the amount of support and supervision provided to participant, to make them more responsible of the activity as time progresses	Does the therapist discuss and agree with participant on the level of intensity of support required to do the tasks and the frequency of next contacts? Is the therapist progressively reducing support (even within the session)? Does the therapist discuss reducing the level of support as programme progresses? Does the therapist use the tapering strategies provided by PrAISED team?		
**Promoting long-term engagement**	The therapist must support the participant to develop intrinsic motivation to ensure that they participants remain active over time	Does the participant seem to enjoy the activity plan? Does it seem that the participants might be able / willing to keep doing the activities over time? Do the therapist work to ensure this, by exploring participant's views?		
**Goal Setting**	The therapist must set goals with the participant that are specific to their interests, functional and active	Does the therapist discuss goal setting with the participant? (sets new goals, review existing goals, adapt/change goals)		

For peer review only

	Does the goal or action plan associated with it lead to the participant doing regular active tasks?		

* Rate as: 1=Visit following principle; 2=visit not following principle; 0=Principle not applicable

For peer review only

For peer review only - http://bmjopen.bmj.com/site/about/guidelines.xhtml

BMJ Open

Protocol for the process evaluation of the Promoting Activity, Independence and stability in early Dementia (PrAISED), following changes required by the COVID-19 pandemic

Journal:	*BMJ Open*
Manuscript ID	bmjopen-2020-039305.R1
Article Type:	Protocol
Date Submitted by the Author:	27-May-2020

Complete List of Authors:	Di Lorito, Claudio; University of Nottingham,<br>Bosco, Alessandro; University of Nottingham - Jubilee Campus, School of Medicine<br>Goldberg, Sarah; University of Nottingham, School of Health Sciences, University of Nottingham<br>Nair, Roshan<br>O'Brien, Rebecca<br>Howe, Louise<br>van der Wardt, Veronika; Philipps-Universitat Marburg<br>Pollock, Kristian; University of Nottingham, School of Health Sciences<br>Booth, Vicky<br>Logan, Phillipa; Community Health Sciences<br>Godfrey, Maureen<br>Dunlop, Marianne<br>Horne, Jane<br>Harwood, Rowan; University of Nottingham School of Health Sciences; Nottingham University Hospitals NHS Trust, Health Care of Older People

**Primary Subject Heading**:	Sports and exercise medicine
Secondary Subject Heading:	Geriatric medicine, Rehabilitation medicine
Keywords:	GERIATRIC MEDICINE, Dementia < NEUROLOGY, REHABILITATION MEDICINE, SPORTS MEDICINE

SCHOLARONE™
Manuscripts

BMJ

I, the Submitting Author has the right to grant and does grant on behalf of all authors of the Work (as defined in the below author licence), an exclusive licence and/or a non-exclusive licence for contributions from authors who are: i) UK Crown employees; ii) where BMJ has agreed a CC-BY licence shall apply, and/or iii) in accordance with the terms applicable for US Federal Government officers or employees acting as part of their official duties; on a worldwide, perpetual, irrevocable, royalty-free basis to BMJ Publishing Group Ltd ("BMJ") its licensees and where the relevant Journal is co-owned by BMJ to the co-owners of the Journal, to publish the Work in this journal and any other BMJ products and to exploit all rights, as set out in our licence.

The Submitting Author accepts and understands that any supply made under these terms is made by BMJ to the Submitting Author unless you are acting as an employee on behalf of your employer or a postgraduate student of an affiliated institution which is paying any applicable article publishing charge ("APC") for Open Access articles. Where the Submitting Author wishes to make the Work available on an Open Access basis (and intends to pay the relevant APC), the terms of reuse of such Open Access shall be governed by a Creative Commons licence – details of these licences and which Creative Commons licence will apply to this Work are set out in our licence referred to above.

Other than as permitted in any relevant BMJ Author's Self Archiving Policies, I confirm this Work has not been accepted for publication elsewhere, is not being considered for publication elsewhere and does not duplicate material already published. I confirm all authors consent to publication of this Work and authorise the granting of this licence.

For peer review only

Protocol for the process evaluation of the Promoting Activity, Independence and stability in early Dementia (PrAISED), following changes required by the COVID-19 pandemic

Claudio Di Lorito, Alessandro Bosco, Sarah Goldberg, Roshan das Nair, Rebecca O'Brien, Louise Howe, Veronika van der Wardt, Kristian Pollock, Vicky Booth, Pip Logan, Maureen Godfrey, Marianne Dunlop, Jane Horne, Rowan H. Harwood

Corresponding author: Claudio Di Lorito, email: claudio.dilorito@nottingham.ac.uk

For peer review only

Abstract

Introduction. The PrAISED Randomised Controlled Trial (RCT) is evaluating a home-based, face-to-face, individually tailored, activity and exercise programme for people living with dementia. Social distancing requirements following the COVID-19 pandemic necessitated rapid changes to intervention delivery.

Methods and analysis. A mixed methods process evaluation will investigate how the changes were implemented and the impact that these have on participants' experience. An *implementation study* will investigate how the intervention was delivered during the pandemic. A *study on the mechanisms of impact and context* will investigate how these changes were experienced by the PrAISED participants, their carers and the therapists delivering the intervention. The study will commence in May 2020.

Ethics and dissemination. The PrAISED RCT and process evaluation have received ethical approval number 18/YH/0059. The ISRCTN Registration Number for PrAISED is 15320670. The PrAISED process evaluation will enable us to understand how distancing and isolation affected participants, their activity and exercise routines, and whether the therapy programme could be continued with remote support. This will be valuable both in explaining trial results, and also contribute to understanding and designing new ways of delivering home-based services and rehabilitation interventions for people with dementia and their carers.

Strengths and limitations of this study

- This study will capture the full range of perspectives, by involving in research participants with dementia, their carers and professionals delivering the intervention;
- This study will gather a holistic picture of the phenomenon, as it uses different methodologies, including quantitative and qualitative data, and data triangulation;

- This study will collect qualitative data at two time points, to capture progress over time;
- The qualitative interviews in this study will be carried out remotely, which could pose barriers to participants with dementia;
- This process evaluation team is not independent of the main trial team and this may generate confirmation bias of study hypotheses

For peer review only

Introduction

Dementia is a neurodegenerative condition characterised by a cluster of symptoms, including memory loss and deterioration of motor skills (1-4). More than 50 million people in the world live with dementia (5). Projections estimate that this number will rise to 130 million people in the next 30 years (5). Dementia presents enormous financial burden (6). In the United Kingdom alone, the cost of health and social care for people with the condition is £50 billion, which will grow to £140 billion by 2040 (5). Keeping physically active has benefits for people with dementia on executive functioning, mobility, activities of daily living, independence, and quality of life (7-25), which have been linked to reduced risk of falls, hospital admissions and health and social care costs.

A number of physical activity and exercise intervention programmes have been developed for people with dementia (15,16). Among these is the Promoting Activity, Independence and Stability in Early Dementia (PrAISED) (26), an intervention to promote activity and independence in people with early dementia or mild cognitive impairment, whose clinical and cost-effectiveness is being evaluated in a five-site Randomised controlled Trial (RCT). So far, 300 participants have been randomised to either a control group (brief falls assessment and advice) or an intervention arm (27). Participants in the intervention arm receive an individually-tailored programme of up to 50 visits at home over a period of 52 weeks from a multidisciplinary team including physiotherapists (PTs), occupational therapists (OTs) and rehabilitation support workers (RSWs) (27). The PrAISED programme comprises: physical exercises (i.e. progressive strength, balance and dual-task); functional activities (i.e. activities of daily living with an element of physical activity, such as going out for food shopping); promotion of inclusion in community life (e.g. through provision of information on physical exercise group classes); risk enablement (assessing, mitigating and agreeing on risks to be taken or avoided); and environmental assessment (27).

The PrAISED RCT includes a process evaluation (28), which aims to describe and quantify intervention delivery, identify the key elements that make the intervention effective and the variables affecting participants motivation to adhere to the programme and remain physically active in the long-term (i.e. beyond the active intervention period). These variables, which have been recently synthesised in a theoretical model (29,30), include the social opportunities linked to exercise, the therapeutic relationship built up with the therapists delivering the intervention, family or carer support, the availability and inclusion of the person in community (physical) activities, the accessibility of the environment (e.g. availability of parks, public transport) and the notion of independence and autonomy (e.g. how, when, and where to exercise).

In March 2020, many of the elements enabling and supporting participants in the PrAISED programme became impossible to deliver due to the pandemic of COVID-19. Measures to slow the spread of the virus were advised and then mandated by governments (31-33). People over 70 years of age, especially those with pre-existing conditions, were told to self-isolate to shield them from increased risk of illness, complications, hospitalisation and mortality (34, 35).

The negative effects that social isolation may have on the health and well-being of older people are well known (36). In people with dementia, there might be additional effects, such

For peer review only - http://bmjopen.bmj.com/site/about/guidelines.xhtml

as a negative impact on functioning, through loss of opportunity to engage with family or in activities outside the home. In order to continue the trial and maintain an element of social contact during this unprecedented time, changes were made to the PrAISED programme intervention delivery (Table 1). There were no changes in training, as all therapists delivering PrAISED were recruited and trained before the amendment to PrAISED. Instead, the therapists were provided with new written guidance on how to deliver the intervention remotely (Appendix 1). The participants who were still receiving the intervention when these changes occurred (March 2020) (n=213) automatically started receiving the amended version of the PrAISED programme. The main change was that participants would not receive visit from therapists at home, as this would place them at risk of contracting the virus. Instead, therapists would continue to support the participants remotely, by telephone or video, in line with the Chartered Society of Physiotherapists has issued guidance (37).

These changes might have important implications on the participants' experience of the intervention. Previous studies have found that face-to-face support from therapists facilitates the creation of a strong therapeutic alliance with the person with dementia, which proves an effective tool for adherence (30). Home visits may facilitate co-production of a programme tailored to the person's needs and aspirations, which is linked to feelings of empowerment and autonomy (38). They may also prove positive for the carers, who, as a result of their caring duties, may incur into a lack of social contact (39,40) and reduced quality of life (41). On the other hand, face-to-face support can increase feelings of dependency among participants, potentially resulting in separation anxiety toward the end of the intervention period (30). From the therapists' perspective, delivering an intervention in the participants' homes can be time consuming. It has been reported in previous process evaluations that adding travelling times on top of the existing workload might thwart job satisfaction (42). The use of remote support might rectify some of these negative experiences.

We aim to extend the process evaluation of the PrAISED (28), to investigate the impact of the changes made to PrAISED. Specifically, the proposed study will respond to the research questions:

- How does staying at home, with no current possibility of receiving face-to-face support from therapists, affect the uptake and retention of a physical activity and exercise programme in participants with dementia? How does it affect their ability to remain independent and their quality of life? Are there ways in which people with dementia can be better supported to remain physically active and independent in these circumstances?
- How are therapists supported to deliver a physical activity and exercise programme remotely to participants with dementia? How does this support affect their confidence and ability to deliver the intervention? Are there ways in which therapists can better supported to deliver the intervention remotely?

Methods and analysis

Based on the assumption that *'if intervention X (i.e. PrAISED) is delivered, the mediating variable(s) (e.g. staying at home, support from therapists available only remotely) affects the*

For peer review only - http://bmjopen.bmj.com/site/about/guidelines.xhtml

For peer review only

way in which outcome Y (e.g. uptake and retention of a physical activity and exercise) will occur', a process evaluation aims to understand how an intervention works (43). It does so by studying the 'implementation of the intervention' (e.g. how the intervention is delivered), the 'mechanisms of impact' (e.g. how participants respond individually to the intervention being delivered) and the 'context' (e.g. the physical and social environment affecting participants' response to the intervention) (43).

This process evaluation will adopt a mixed-methods approach, including quantitative data and data ensuing from qualitative interviews. It will consist of two studies: an implementation study and a study on mechanisms of impact and context (Figure 1). The study will commence in May 2020 and the final results are expected to be available in May 2021.

Patient and Public Involvement

The process evaluation study team includes two patient and public involvement (PPI) contributors (MG and MD), who have been involved in the development of the process evaluation and its protocol (also acting as co-authors). The PPI contributors co-designed with the main researcher (CDL) the topic guide for the qualitative interviews of participants with dementia and their carers (see details in "study of mechanisms of impact and context – data collection") and will be involved as co-raters in the qualitative analysis of the transcripts of the interviews (see details in "study of mechanisms of impact and context – data analysis") and in disseminating research findings (e.g. through attending conferences, public dissemination events and co-authoring results' papers).

Implementation study

The study on implementation will investigate how the PrAISED intervention is delivered, following changes in procedure in response to the COVID-19 pandemic. It will focus on four domains (Table 2):

- Fidelity (i.e. the consistency of delivery of PrAISED with the amended protocol);
- Adaptations (i.e. alterations made to delivery of PrAISED to achieve better contextual fit);
- Dose (i.e. how much PrAISED intervention is delivered);
- Reach (i.e. the number of therapists trained to deliver PrAISED and of participants who receive the intervention).

Participants

The implementation study will include participants with dementia in the intervention group, their carers and therapists who are involved in the PrAISED main trial at the time of recruitment (May 2020).

For peer review only - http://bmjopen.bmj.com/site/about/guidelines.xhtml

Data collection

From the participants with dementia:

- Adherence to intervention as per instructions (Fidelity), investigated through qualitative interviewing;
- Adherence to advised activity levels (Dose), investigated through minutes of PrAISED activity per week as recorded on a self- (or carer-) completed monthly calendar;
- The extent to which participants with dementia come into contact with the intervention (Reach), investigated by totalling the number of participants who completed the programme;
- Alterations that participants made to achieve better contextual fit (Adaptations), investigated through qualitative interviewing.

From the therapists:

Evaluation of the delivery of the adapted intervention, including:

- Number and length of remote sessions the therapists have with participants (dose and reach): A record of the date, length in minutes, and therapist type (PT, OT and RSW) will be recorded for each contact. The information is collated by the research team each week.
- Goals set for participants (adaptations): Goals that have been set with the participants are documented by the therapists and collated centrally by the research team.
- Intervention content (fidelity, adaptations): One intervention session provided remotely by each therapist will be audio-recorded. To ensure safe handling and storing of sensitive data, the session between the therapist and the participant will be recorded remotely by one researcher within the PrAISED team with an encrypted digital audio recorder.

Data analysis

The data from the implementation study will be analysed using IBM SPSS Statistics version 26 (44). Descriptive statistical analysis will be used to measure fidelity, dose and reach.

The audio recordings will be transferred onto an encrypted and password protected university computer server. The content will be assessed independently by two raters against 14 core principles set out in the PrAISED therapists' training manual (i.e. *'visit following core principle', 'visit not following core principle', 'Principle not applicable'*). An audio-analysis template will list the core principles, provide operational definitions of each of them, accompanied with practical examples of the application of principle, to facilitate retrieval of content during analysis (Appendix 2).

For peer review only - http://bmjopen.bmj.com/site/about/guidelines.xhtml

Prior to independent audio analysis, the two raters will pilot-test the rating procedure using a sample audio recording, to check inter-rater reliability. Scores from the two raters will be compared to determine inter-rater reliability, and if inconsistency arises in scoring, consensus will be reached through discussion between the two raters or through involvement of a third rater.

Study on mechanisms of impact and context

The study on mechanisms of impact and context will investigate the participants and therapists' experience of the intervention, and any variable mediating intervention outcomes (e.g. social distancing).

Participants

For each research site, we will include:

1. Participants with dementia and their carer, further divided in:

- Intervention arm (i.e. receiving the active intervention);
- Control arm (i.e. receiving treatment as usual, included to investigate whether there are any relevant differences between control and intervention arm);
- Those who have withdrawn from the therapy programme, if they agree to be interviewed.

Purposive sampling will be carried out to ensure a diverse and representative sample in relation to gender, ethnicity, residence status (i.e. living independently or living with carer) and the different research sites involved in PrAISED (i.e. Nottinghamshire, Derbyshire, Lincolnshire, Somerset and Oxfordshire). The main researcher (CDL) will access the PrAISED RCT database and select participants from the different subgroups.

We will not exclude participants who do not have mental capacity to agree to participate or who show fluctuating capacity at the point of the interview, for the following reasons: Firstly, they might still provide precious insight into the mechanisms of the intervention; secondly, their (fluctuating) cognition may have an impact and affects their response toward the intervention; finally, from an ethical standpoint, we aim to give voice to all those whose life is primarily affected by our research. However, we will take into account capacity to give consent (or lack thereof) during the course of the interview, by relying, for example, on different degrees of carer support during the session.

2. Therapists will be purposively sampled to be involved in the process evaluation. The main researcher (CDL) will access the PrAISED RCT database and select therapists from the different professions (i.e. physiotherapists, occupational therapists and rehabilitation support workers) and research sites.

For peer review only - http://bmjopen.bmj.com/site/about/guidelines.xhtml

In line with Guest, Bunce and Johnson (45), we argue that, given the lack of guidance around reaching data saturation, there is a need to adopt appropriate 'tests of adequacy' for sample sizes in qualitative research. Based on the notion of 'conceptual density' (i.e. gathering data until a *sufficient depth* of understanding of the domains under investigation is reached) (46), we will adopt a *Conceptual Depth Scale* developed by Nelson (46) (Table 3), which assigns a score ranging from 1 (low) to 3 (high) to establish whether conceptual density is reached in relation to:

- *'Range'* (e.g. extent of diversity of data sources);
- '*Complexity'* (e.g. extent of networks / links across data);
- '*Subtlety'* (e.g. extent of similarity across data);
- *'Validity'* (e.g. extent to which data are transferable to other settings)

The scoring will be performed by two researchers independently of each other. The scale is used as instrument to check whether consensus is reached among researchers with respect to data saturation, rather than as quantitative assessment to determine a saturation point for data interpretation. We anticipate that conceptual density will be reached by inclusion of up to 20 participants with dementia (and 20 carers), and 20 therapists across all research sites.

Data collection

The investigation of the mechanisms of impact and context will be based on qualitative interviews with participants. The first interview will be conducted one month following the change of intervention in response to the COVID-19 pandemic (i.e. May 2020). Follow up interviews will be considered, if the measures imposed following the COVID-19 pandemic are still in place, to monitor progress over time.

The interviews will consist of:

- Remote interviews (different options will be offered, including telephone or video calling, depending on participants' preference) with participants with dementia and their carers (as a dyad, so that the carer can provide information, as well as support, if needed). We will use a speaker phone for everyone to be able to contribute. Prior to the session, the researcher will mail (or email) a copy of the consent form. A verbal consent for both the participant with dementia and the carer will be recorded on tape, before the interview begins.

- Remote interviews with therapists (i.e. occupational therapist, physiotherapist and rehabilitation support worker). Verbal consent will be recorded on tape prior to the interview.

The topic guide for the qualitative interviews is informed by the *PHYT in dementia* (PHYsical activity behaviour change Theory in dementia), whose development and validation we reported elsewhere (29,30). Through this theoretical framework, we identified potential variables mediating intervention outcomes and developed several prompts to stimulate

For peer review only - http://bmjopen.bmj.com/site/about/guidelines.xhtml

discussion. Exploration of context will include the impact of isolation, and its effects on exercise, activity and mental well-being.

We developed the topic guides as a collaborative effort between the research team and the PPI contributors, who helped to ensure that the interview prompts are relevant, meaningful and accessible for the participants. Although questions are study-specific, the prompts are broad in scope, to ensure that the participants feel free to express their ideas around unanticipated causal processes and consequences. The participants may also raise additional topics and issues which they feel are particularly relevant in the context of the COVID-19 pandemic, and these will be explored accordingly.

The qualitative interviews are expected to last around 40 minutes, depending on participants' engagement in the process, their cognitive abilities, and logistics.

Data analysis

Data will be analysed through framework analysis (47). This method is ideal in social and health care qualitative research studies with large data sets. Framework analysis will ensure in-depth exploration of data, a transparent audit trail of the process of analysis, and the understanding of data interpretation (e.g. a description of how data link to each other and according to the objective of the study) through visual mapping (47).

Data analysis will follow the steps for good practice in Framework Analysis identified by Gale et al. (47):

1. *Verbatim transcription* of the interviews by a professional transcriber, who will also anonymise data. Large margins and double line spacing in the transcripts will be left to create room for coding and note taking.
2. *Familiarisation with the transcripts* by the main researcher (CDL), who will write down analytical notes on margins.
3. *Coding of a sample of three transcripts* by the main researcher, a second researcher within the research team and one PPI contributor, who will independently underline relevant pieces of text and write coding labels for each, reflecting the constructs included in the topic guide. However, to prevent the omission of important data, if novel constructs are identified from the transcripts, new coding labels will be generated.
4. *Development of a working analytical framework* through team work of the three coders, who will create a set of initial codes through synthesis of individual coding and operational definitions. Two more transcripts will be coded by two coders to check whether the initial working analytical framework is suitable. Eventually, a stable set of codes, clustered into umbrella categories will be identified.
5. *Use of the working analytical framework* by the main researcher (CDL) to code the whole set of transcripts in NVivo 12 (48). Double coding will be conducted by another researcher.

For peer review only - http://bmjopen.bmj.com/site/about/guidelines.xhtml

6. *Charting of data into the framework matrix* by the main researcher on NVivo. The matrix will map out codes (one per column) and participants (one per row). The relevant quotes will be transferred from NVivo onto the matrix.
7. *Interpretation of data* by the main researcher, who will develop themes from the matrix by making connections within and between participants and categories. This will be an iterative process, with regular review from members of the research team.

Ethics and dissemination

The PrAISED trial and process evaluation have received ethical approval number 18/YH/0059. The ISRCTN Registration Number for PrAISED is 15320670.

This protocol, grounded in the MRC framework for process evaluation of complex intervention (43), outlines the rationale, design and methods for the process evaluation of the Promoting Activity, Independence and Stability in Early Dementia and mild cognitive impairment (PrAISED), following the changes made as a result of the restrictions on face-to-face contact during the COVID-19 pandemic.

In only a few months, the COVID-19 pandemic has required dramatic changes to our lifestyles and caused unprecedented operational strain on national health and social care systems. There is a need for scientific evidence to inform research and services in response to the current challenges, as well as preparation for services after the pandemic and potential future events. In these respects, the final process evaluation report, which will be disseminated in scientific journals and to the public (e.g. through public engagement events), will report on the impact that the social distancing measures introduced in PrAISED have had on research participants and therapists. By comparing the evidence gathered through this study with the original PrAISED process evaluation (28) and the wider literature, this process evaluation will contribute knowledge on ways in which individuals belonging to the most vulnerable groups in society can be better supported and motivated to remain physically active and healthy in their homes without face-to-face support. In addition, by triangulating data from this process evaluation with some quantitative measures from the RCT (e.g. quality of life (QoL) and carer strain), we will be able to gather a more comprehensive picture of the impact that the COVID-19 has had on the lives of participants.

This work will also present important implications in theory advancement. Our dissemination plans include a paper further validating the *PHYT in dementia*, the behaviour change theoretical model that our research team previously developed and validated through data from the original PrAISED process evaluation (29,30). Results from this work will contribute further evidence to confirm / challenge the validity of the model in explaining motivation to be physically active, in the context of social distancing. Finally, based on findings from this process evaluation, we aim to develop a methodological paper outlining strategies that can be used to involve research participants remotely in an ethical, meaningful and practically feasible way. This model can be refined through input from research teams conducting rehabilitation studies in similar circumstances, such as the FinCH study (49), to derive a research platform that can be shared to inform / guide good practice in future research.

For peer review only

In conclusion, this process evaluation represents one of the first efforts to document how an ongoing research programme was adapted as a result of the COVID-19 pandemic. This study will support the critical reflection by the PrAISED team on positive and negative aspects of these adaptations. It will also provide transferable information to develop strategies to effectively deliver rehabilitation remotely, in the presence of extraordinary circumstances (e.g. social distancing and staying at home).

Funding statement: This protocol presents independent research funded by the United Kingdom National Institute for Health Research (NIHR) under its Programme Grants for Applied Research funding scheme (RP-PG-0614-20007). The views expressed are those of the authors and not necessarily those of the National Health Service, the NIHR or the Department of Health and Social Care.

Conflict of interest: None to declare

Authors' contributions: CDL contributed to the conception and design of the study, and the development of all the elements of the process evaluation. AB contributed to the planning of the process evaluation, the analysis plan for therapists' audio recordings, and provided feedback and final approval of the manuscript. SG contributed the PrAISED RCT information, helped in the conception of the study, and provided feedback and final approval of the manuscript. RdN contributed to the development of the analysis plan for the qualitative element of the study and the therapists' audio recordings, and provided feedback and final approval of the manuscript. ROB contributed to develop the study design and the analysis plan of the therapists' audio recordings, and provided feedback and final approval of the manuscript. LH helped to develop the topic guide for the therapists' qualitative interviews, as well as the analysis plan, and provided feedback and final approval of the manuscript. VvdW contributed to the study conception and design, the implementation study and the quantitative data analysis plan, and provided feedback and final approval of the manuscript. KP provided guidance and expertise on the development of the qualitative interviews for participants with dementia and carers, and provided feedback and final approval of the manuscript. VB contributed to the design and analysis plan of the implementation study, and provided feedback and final approval of the manuscript. PL contributed to the discussion section of the manuscript, and provided feedback and final approval of the manuscript. MG and MD were the PPI collaborators of the study. They contributed to the development of the topic guide for the qualitative interviews of the participants with dementia and their carers, and provided feedback and final approval of the manuscript. JH contributed to the discussion section of the manuscript, and provided feedback and final approval of the manuscript. RHH contributed the PrAISED RCT information, helped in the conception of the study and provided feedback and final approval of the manuscript.

Words: 3525

Figure 1. Method of Process Evaluation

For peer review only - http://bmjopen.bmj.com/site/about/guidelines.xhtml

For peer review only

Reference

(1). Martyr, A. and L. Clare, Executive function and activities of daily living in Alzheimer's disease: a correlational meta-analysis. Dementia and geriatric cognitive disorders, 2012. 33(2-3): p. 189-203.

(2). Giebel, C.M., et al., Deterioration of basic activities of daily living and their impact on quality of life across different cognitive stages of dementia: a European study. International Psychogeriatrics, 2014. 26(8): p. 1283-1293.

(3). Giebel, C.M., C. Sutcliffe, and D. Challis, Activities of daily living and quality of life across different stages of dementia: a UK study. Aging & Mental Health, 2015. 19(1): p. 63-71.

(4). Alzheimer's Research UK (2019). About dementia. Available online: https://www.alzheimersresearchuk.org/about-dementia/?gclid=Cj0KCQjw3JXtBRC8ARIsAEBHg4myuYeahFMLCcSGLr-flQxznmdB-dObW2gXc5MUN9o_dfNw5wwI5EwaAvH6EALw_wcB (accessed on 26 March 2020).

(5). Alzheimer's Society (2020). Facts for the Media. Available online: https://www.alzheimers.org.uk/about-us/news-and-media/facts-media (accessed on 26 March 2020).

(6). Lewis F, Karlsberg Schaffer S, Sussex J, O'Neill P, Cockcroft L. The trajectory of dementia in the UK-making a difference. Office of Health Economics Consulting Reports. 2014 Jun 1.

(7). Forbes D, Forbes SC, Blake CM, Thiessen EJ, Forbes S. Exercise programs for people with dementia. Cochrane Database of Systematic Reviews. 2015(4). doi:10.1002/14651858.CD006489.pub4.

(8). Potter R, Ellard D, Rees K, Thorogood M. A systematic review of the effects of physical activity on physical functioning, quality of life and depression in older people with dementia. International journal of geriatric psychiatry. 2011 Oct;26(10):1000-11.

(9). Blankevoort CG, Van Heuvelen MJ, Boersma F, Luning H, De Jong J, Scherder EJ. Review of effects of physical activity on strength, balance, mobility and ADL performance in elderly subjects with dementia. Dementia and geriatric cognitive disorders. 2010;30(5):392-402.

(10). Heyn P, Abreu BC, Ottenbacher KJ. The effects of exercise training on elderly persons with cognitive impairment and dementia: a meta-analysis. Archives of physical medicine and rehabilitation. 2004 Oct 1;85(10):1694-704.

(11). Hauer K, Schwenk M, Zieschang T, Essig M, Becker C, Oster P. Physical training improves motor performance in people with dementia: a randomized controlled trial. Journal of the American Geriatrics Society. 2012 Jan;60(1):8-15.

For peer review only

(12). Hoffmann K, Sobol NA, Frederiksen KS, Beyer N, Vogel A, Vestergaard K, Brændgaard H, Gottrup H, Lolk A, Wermuth L, Jacobsen S. Moderate-to-high intensity physical exercise in patients with Alzheimer's disease: a randomized controlled trial. Journal of Alzheimer's Disease. 2016 Jan 1;50(2):443-53.

(13). Lamb SE, Sheehan B, Atherton N, Nichols V, Collins H, Mistry D, Dosanjh S, Slowther AM, Khan I, Petrou S, Lall R. Dementia And Physical Activity (DAPA) trial of moderate to high intensity exercise training for people with dementia: randomised controlled trial. bmj. 2018 May 16;361:k1675.

(14). Lowery, D., Cerga-Pashoja, A., Iliffe, S., Thuné-Boyle, I., Griffin, M., Lee, J., Bailey, A., Bhattacharya, R. and Warner, J., 2014. The effect of exercise on behavioural and psychological symptoms of dementia: the EVIDEM-E randomised controlled clinical trial. *International journal of geriatric psychiatry*, *29*(8), pp.819-827.

(15). Pitkälä KH, Pöysti MM, Laakkonen ML, Tilvis RS, Savikko N, Kautiainen H, Strandberg TE. Effects of the Finnish Alzheimer disease exercise trial (FINALEX): a randomized controlled trial. JAMA internal medicine. 2013 May 27;173(10):894-901.

(16). Prick AE, De Lange J, Scherder E, Twisk J, Pot AM. The effects of a multicomponent dyadic intervention with physical exercise on the cognitive functioning of people with dementia: A randomized controlled trial. Journal of aging and physical activity. 2017 Oct 1;25(4):539-52.

(17). Rolland Y, Pillard F, Klapouszczak A, Reynish E, Thomas D, Andrieu S, Rivière D, Vellas B. Exercise program for nursing home residents with Alzheimer's disease: a 1-year randomized, controlled trial. Journal of the American Geriatrics Society. 2007 Feb;55(2):158-65.

(18). Schwenk M, Zieschang T, Oster P, Hauer K. Dual-task performances can be improved in patients with dementia: a randomized controlled trial. Neurology. 2010 Jun 15;74(24):1961-8.

(19) Schwenk M, Zieschang T, Englert S, Grewal G, Najafi B, Hauer K. Improvements in gait characteristics after intensive resistance and functional training in people with dementia: a randomised controlled trial. BMC geriatrics. 2014 Dec;14(1):73.

(20). Suzuki T, Shimada H, Makizako H, Doi T, Yoshida D, Tsutsumimoto K, Anan Y, Uemura K, Lee S, Park H. Effects of multicomponent exercise on cognitive function in older adults with amnestic mild cognitive impairment: a randomized controlled trial. BMC neurology. 2012 Dec 1;12(1):128.

(21). Telenius EW, Engedal K, Bergland A. Effect of a high-intensity exercise program on physical function and mental health in nursing home residents with dementia: an assessor blinded randomized controlled trial. PloS one. 2015;10(5).

(22). Forbes D, Forbes SC, Blake CM, Thiessen EJ, Forbes S. Exercise programs for people with dementia. Cochrane Database of Systematic Reviews. 2015(4).

(23). Potter R, Ellard D, Rees K, Thorogood M. A systematic review of the effects of physical activity on physical functioning, quality of life and depression in older people with dementia. International journal of geriatric psychiatry. 2011 Oct;26(10):1000-11.

For peer review only

(24). Blankevoort CG, Van Heuvelen MJ, Boersma F, Luning H, De Jong J, Scherder EJ. Review of effects of physical activity on strength, balance, mobility and ADL performance in elderly subjects with dementia. Dementia and geriatric cognitive disorders. 2010;30(5):392-402.

(25). Heyn P, Abreu BC, Ottenbacher KJ. The effects of exercise training on elderly persons with cognitive impairment and dementia: a meta-analysis. Archives of physical medicine and rehabilitation. 2004 Oct 1;85(10):1694-704.

(26). Booth V, Harwood RH, Hood-Moore V, Bramley T, Hancox JE, Robertson K, Hall J, Van Der Wardt V, Logan P. Promoting Activity, Independence and Stability in Early Dementia and Mild Cognitive Impairment (PrAISED): Development of an Intervention for People with Mild Cognitive Impairment and Dementia. Clinical Rehabilitation 2018; 32: 855-864. doi.org/10.1177/0269215518758149

(27). Bajwa RK, Goldberg SE, Van der Wardt V, Burgon C, Di Lorito C, Godfrey M, Dunlop M, Logan P, Masud T, Gladman J, Smith H. A randomised controlled trial of an exercise intervention promoting activity, independence and stability in older adults with mild cognitive impairment and early dementia (PrAISED)-A Protocol. Trials. 2019 Dec;20(1):1-1.

(28). Di Lorito C, Pollock K, Harwood R, das Nair R, Logan P, Goldberg S, Booth V, Godfrey M, Dunlop M, Van der Wardt V. Protocol for the process evaluation of the promoting activity, independence and stability in early dementia and mild cognitive impairment (PrAISED 2) randomised controlled trial. Maturitas. 2019 Apr 1;122:8-21.

(29). Di Lorito C, Pollock K, Harwood R, das Nair R, Logan P, Goldberg S, Booth V, Vedhara K, Van Der Wardt V. A scoping review of behaviour change theories in adults without dementia to adapt and develop the 'PHYT in dementia', a model promoting physical activity in people with dementia. Maturitas. 2019 Mar 1;121:101-13.

(30). Di Lorito C, Bosco A, Pollock K, H Harwood R, Das Nair R, Logan P, Goldberg S, Booth V, Vedhara K, Godfrey M, Dunlop M. External Validation of the 'PHYT in Dementia', a Theoretical Model Promoting Physical Activity in People with Dementia. International Journal of Environmental Research and Public Health. 2020 Jan;17(5):1544.

(31). World Health Organization (2020). WHO Director-General's opening remarks at the media briefing on COVID-19 - 11 March 2020. Retrieved from: https://www.who.int/dg/speeches/detail/who-director-general-s-opening-remarks-at-the-media-briefing-on-covid-19---11-march-2020 (accessed: 02 April 2020).

(32). Ferguson NM, Laydon D, Nedjati-Gilani G, Imai N, Ainslie K, Baguelin M, Bhatia S, Boonyasiri A, Cucunubá Z, Cuomo-Dannenburg G, Dighe A. Impact of Non-Pharmaceutical Interventions (NPIs) to Reduce COVID-19 Mortality and Healthcare Demand (16 March 2020). URL https://doi. org/10.25561/77482.

(33). Sun P, Lu X, Xu C, Sun W, Pan B. Understanding of COVID-19 based on current evidence. Journal of medical virology. 2020 Feb 25.

(34). Public Health England (2020). Guidance on social distancing for everyone in the UK. Available at: https://www.gov.uk/government/publications/covid-19-guidance-on-social-

For peer review only - http://bmjopen.bmj.com/site/about/guidelines.xhtml

distancing-and-for-vulnerable-people/guidance-on-social-distancing-for-everyone-in-the-uk-and-protecting-older-people-and-vulnerable-adults (Accessed: 24 March 2020)

(35). Zu ZY, Jiang MD, Xu PP, Chen W, Ni QQ, Lu GM, Zhang LJ. Coronavirus disease 2019 (COVID-19): a perspective from China. Radiology. 2020 Feb 21:200490.

(36). Hawton A, Green C, Dickens AP, Richards SH, Taylor RS, Edwards R, Greaves CJ, Campbell JL. The impact of social isolation on the health status and health-related quality of life of older people. Quality of Life Research. 2011 Feb 1;20(1):57-67.

(37) The Chartered Society of Physiotherapy. COVID-19: guide for rapid implementation of remote consultations. Retrieved from: https://www.csp.org.uk/publications/covid-19-guide-rapid-implementation-remote-consultations Accessed on 09 April 2020.

(38). Bosco A, Schneider J, Coleston-Shields DM, Orrell M. Dementia care model: Promoting personhood through co-production. Archives of gerontology and geriatrics. 2019 Mar 1;81:59-73.

(39). Brodaty H, Hadzi-Pavlovic D. Psychosocial effects on carers of living with persons with dementia. Australian & New Zealand Journal of Psychiatry. 1990 Sep;24(3):351-61.

(40). Graham C, Ballard C, Sham P. Carers' knowledge of dementia, their coping strategies and morbidity. International journal of geriatric psychiatry. 1997 Sep;12(9):931-6.

(41). Gaugler JE, Kane RL, Kane RA, Newcomer R. Unmet care needs and key outcomes in dementia. Journal of the American Geriatrics Society. 2005 Dec;53(12):2098-105.

(42). Bosco A, Paulauskaite L, Hall I, Crabtree J, Soni S, Biswas A, Cooper V, Poppe M, King M, Strydom A, Crawford MJ. Process evaluation of a randomised controlled trial of PBS-based staff training for challenging behaviour in adults with intellectual disability. PloS one. 2019;14(8).

(43). Moore GF, Audrey S, Barker M, Bond L, Bonell C, Hardeman W, Moore L, O'Cathain A, Tinati T, Wight D, Baird J. Process evaluation of complex interventions: Medical Research Council guidance. bmj. 2015 Mar 19;350.

(44). IBM Corp. Released 2017. IBM SPSS Statistics for Windows, Version 25.0. Armonk, NY: IBM Corp.

(45). Guest G, Bunce A, Johnson L. How many interviews are enough? An experiment with data saturation and variability. Field methods. 2006 Feb;18(1):59-82.

(46). Nelson J. Using conceptual depth criteria: addressing the challenge of reaching saturation in qualitative research. Qualitative research. 2017 Oct;17(5):554-70.

(47). Gale NK, Heath G, Cameron E, Rashid S, Redwood S. Using the framework method for the analysis of qualitative data in multi-disciplinary health research. BMC medical research methodology. 2013 Dec;13(1):117.

(48). NVivo qualitative data analysis Software (2018). Melbourne, Australia: QSR International Pty Ltd.

For peer review only

For peer review only - http://bmjopen.bmj.com/site/about/guidelines.xhtml

(49). Logan P, McCartney K, Armstrong S, Clarke A, Conroy S, Darby J….& Horne J. Evaluation of the Guide to Action Care Home fall prevention programme in care homes for older people: protocol for a multi-centre, single blinded, cluster randomised controlled trial (FinCH). East Midlands Research into Ageing Network (EMRAN) Discussion Paper Series Issue 25 Feb 2019. ISSN 2059-3341.

For peer review only

For peer review only - http://bmjopen.bmj.com/site/about/guidelines.xhtml

Table 1. Main changes made to the PrAISED intervention, compared to the original version (26,27)

Delivery of intervention	Provision of support to the therapists
The therapists were provided with written guidance on how to deliver the intervention (Appendix 1)	Increased access to:<br>• Monthly teleconferences across all sites;<br>• Teleconferences at individual sites;<br>• Provision of a regularly updated list of resources;<br>• Provision of informal support through email and phone;<br>• Provision of information and support tailored to the situation and change in practice

For peer review only - http://bmjopen.bmj.com/site/about/guidelines.xhtml

Table 2. Implementation study

	Delivery (Therapists)	*Adherence (Participants)*
Fidelity	Delivery of intervention against PrAISED 2 principles (through audio content)	Adherence to intervention as per instructions (through interview)
Dose	Frequency and length of contact sessions with participant*	Minutes per week recorded on calendar*
Reach	Number of contact sessions with participant*	Number of participants who completed the programme*
Adaptations	Adaptations made to deliver the sessions (through interview)	Adaptations that participants made to physical activity and exercise (through interview)

* Data gathered during main Trial

For peer review only - http://bmjopen.bmj.com/site/about/guidelines.xhtml

Table 3. Conceptual Depth Scale (46)

Criteria (with sources of evidence)	Low (1)	Medium (2)	High (3)
Range (e.g. frequency and variety of codes; multiplicity of data sources)	Few examples to sup-port concepts. Only a single data-type	→	Abundant examples to sup-port concepts. Multiple data-types
Complexity (e.g. coding trees; positional maps; matrices)	Descriptive codes; simple or basic connections between codes; low level analysis	→	Sophisticated networks; abstract conceptual categories which synthesise a range of codes and concepts
Subtlety (e.g. memos; social worlds diagrams)	Conceptual language is regarded as unproblematic and one dimensional	→	Conceptual language is understood as rich, ambiguous and multi-dimensional
Resonance (literature)	Weak resonance; emerging theory is re-mote from existing literature and theoretical frameworks	→	Strong resonance; emerging theory makes sense along-side existing literature; there are correlations with other theoretical frameworks, albeit with variations and novel-ties
Validity (e.g. applicability test)	Low level theorising and inward facing; the findings have limited application to the re-search participants or those familiar with similar contexts.	→	Abstract level theorising and outward facing; the findings make sense to those in the social context of the re-search, or ones broadly similar.

For peer review only - http://bmjopen.bmj.com/site/about/guidelines.xhtml

Figure 1. Method of Process Evaluation

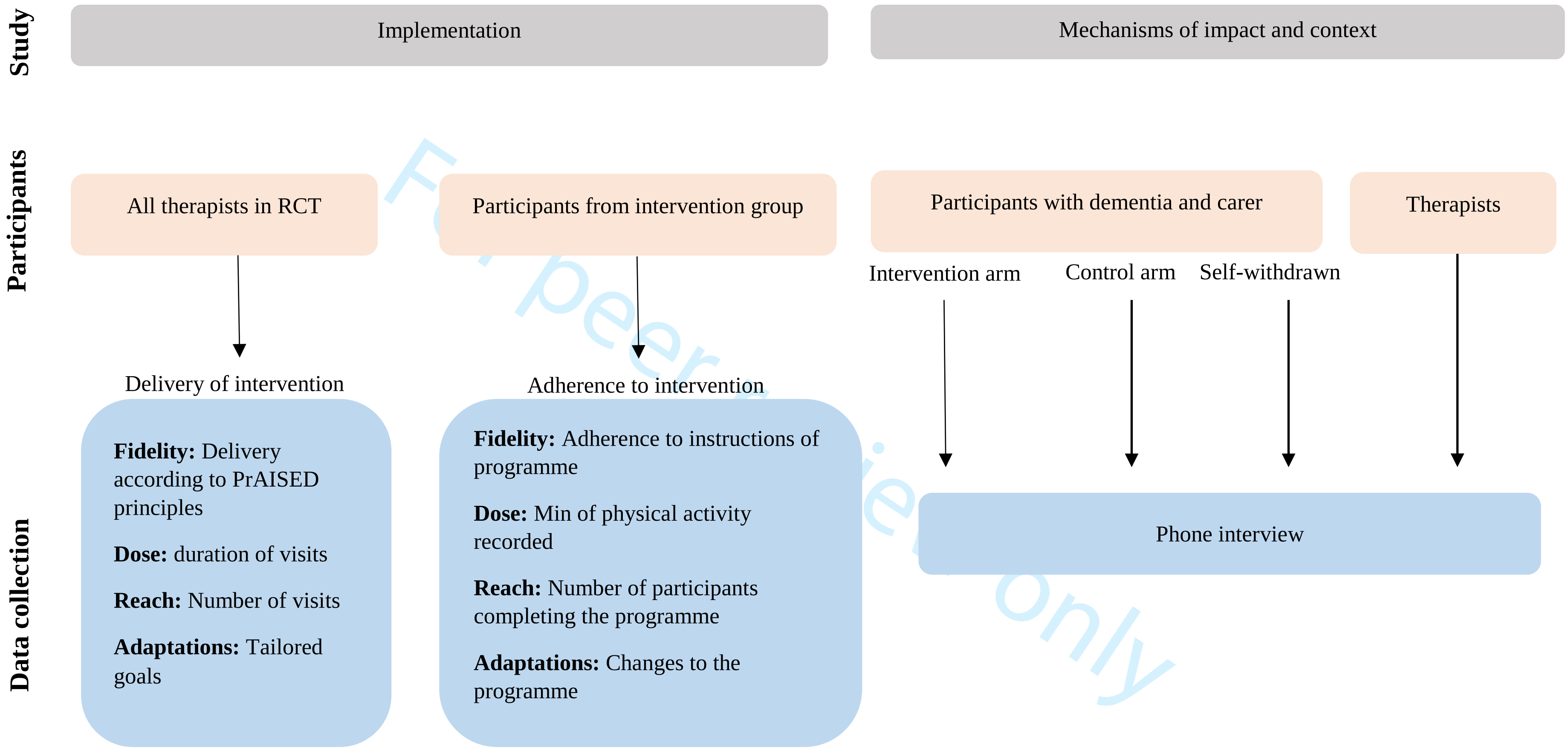

For peer review only

Appendix 1. Guidance distributed among the PrAISED therapists on the changes made to the intervention

Plan for PrAISED2 Intervention in response to COVID19 restrictions (18.03.2020)

Immediate plan

The NIHR have stated that their funded studies should stop all non-essential face to face contact. The PrAISED intervention is not considered essential care and therefore we must stop all face to face contact with our participants.

However, because we have a duty of care to our patients considering many of them will be following the governments advice to reduce all social contact, we have devised a contingency plan to continue with the PrAISED intervention.

Intervention Group Participants

Therapy teams should contact all participants currently in the trial, or their carers if more appropriate, to explain the change in practice as below.

On-going Intervention Group Participants

Visits to participants should be replaced with **telephone coaching** as per their normal schedule, in terms of frequency. For example, if you are seeing someone weekly, this should be continued until they reach the time to reduce to fortnightly. This is the example frequency schedule set out in the intervention manual, however, continue to adapt this as appropriate in the same way you have been doing.

- Month 1-2: bi-weekly
- Month 3-6: weekly
- Month 6-9: fortnightly
- Month 9-12: monthly

The length of the phone call may be much shorter depending on what is discussed.

The content of the phone call should be guided by the telephone coaching instructions below.

Some participants won't be suitable for telephone calls. If the participant is unable to engage with telephone coaching the carer should be contacted to determine if they may be able to use the telephone coaching to support the participant. If the telephone coaching is of no benefit to either the participant or the carer, then a courtesy telephone call should be given each month to keep in touch with the carer or participant as appropriate.

Final sessions should be carried out via the telephone as appropriate; these should be followed up with an end of therapy letter and any follow up material being provided using the post or email if appropriate.

For peer review only

New Intervention Group Participants

Intervention group participants seen by the research team but not yet seen by therapy team, or who are in the assessment phase of the intervention, should be informed that they are not going to receive the PrAISED intervention until the current restrictions are lifted.

Control Group Participants

If you have completed the first control visit you can carry out up to two follow up visits by telephone as per the guidance below. If the first control visit has not yet been completed, please inform the participant that they are not going to receive the PrAISED intervention until the current restrictions are lifted.

Therapy Visit Log

Continue to complete the therapy visit log, via the hyperlink, for all telephone calls. Please put telephone coaching in the comments box.

Medium-Term Plan

It is expected that PrAISED therapy staff at each site will deliver the immediate plan outlined above.

However, as the situation changes a medium-term plan (outlined below) may come into action.

If sites cannot deliver the telephone coaching sessions due to therapy staffing difficulties, the university staff may have capacity to be able to support. The PI from each site must contact the University as soon as possible if this happens. For university staff to be able to do the telephone coaching sessions effectively, we will need to know:

- the participant's details (e.g., contact telephone number for them and the carer/informant)
- a synopsis of the previous intervention session and what they are currently working on

As each site is using different participant documentation systems, the PIs should liaise with Sarah Goldberg or Rebecca O'Brien, to form a contingency plan on how this will happen and how information is to be transferred and stored.

Telephone Coaching Instructions

Before making the telephone call make sure you have looked at NHS England current advice for the client group you are dealing with, as this is likely to change on a regular basis (https://www.nhs.uk/conditions/coronavirus-covid-19/). Participants may have concerns about their current situation that need answering before the participant will engage in coaching.

- Explain who you are and why you're calling.

For peer review only

- Ask how they are and discuss any immediate concerns (they may need signposting as appropriate).
- Review their current activity and exercise plan.
- Review what they are currently doing during their day.
- Be aware that for many participants all their activities may have stopped.
- Form a plan of what they can do within the **current** restrictions. For example, currently people are advised it is ok to walk outside as long as they stay 2m away from other people.
- Help them to make a daily plan of activities. For example, doing exercises more frequently, or if they are no longer walking outside can they walk in the garden or up and down the stairs to get some cardiovascular exercise.
- Advise against sitting for long periods of time. For example, use a timer to remind yourself to get up or get up during advert breaks in television programmes.
- If the person is able to and wants to, they could put you on speaker phone while you go through their exercise programme with them. Only do this if they have the capacity to do this with their telephone. This could also be done with their carer or family member or named informant.
- Be aware people may be feeling quite worried and/or low in mood. You may need to discuss the benefits of, and encourage them to continue to carry out daily activities or routines, such as getting dressed, or taking meals on time.
- Participants may raise safeguarding issues such as identifying they are low on medication and there is no one to help them with this. This will need to addressed using the usual safeguarding procedures.
- If participants are complaining of COVID 19 symptoms they should be encouraged to follow the current advice from NHS direct or to phone 111.

It is expected these telephone coaching guidelines will evolve as PrAISED therapists start conducting these sessions. Guidance can come from outside sources, e.g., RCOT have recently shared this online https://www.rcot.co.uk/staying-well-when-social-distancing. It is important that we share good practice and suggestions and will discuss these guidelines during our PrAISED Therapist Teleconferences.

For peer review only

Appendix 2. Template for analysis of audio-recordings of therapists' remote sessions

Principle	Description	Examples	Rater 1*	Rater 2*
**Intensive**	Physical activity must be performed for at least 150 minutes per week. Participants may require more or less intensive support to achieve this.	Does therapist ask about activity times or amount of activity done over the last week? Do they discuss and agree with participant level of intensity of support required and frequency of next visits? Do they discuss activity plans for the upcoming week?		
**Tailored**	The therapist must work with participant to select and tailor physical exercise / activities that will be of most benefit and interest	Does the therapist make the participant feel they are in control of the activities to be done? For example do they ask whether the participant wants to do the activity? Does the participant seem to enjoy doing it? Is the participant given choices around exercise/activity? Does the therapist make recommendations on activity/exercise based on what the participant has said, or what they have observed the participant do?		
**Challenging**	The tasks must be challenging	Are the tasks challenging enough for participant, but still within their capabilities (i.e. realistically achievable)?		
**Progressive**	The tasks must be progressive	Is the therapist increasing the challenge of the task progressively (even within the same session)? Do they discuss progressing the tasks, now or in the future?		
**Promoting / improving independence**	The tasks must promote or improve independence (ability to complete tasks without dependence on others)	Is the therapist asking the participant to carry out activities independently or working towards them being independent? (e.g. personal, domestic or leisure ADLs, navigating the kitchen, making tea). Do they		

For peer review only

		discuss how the participant could be more independent or set goals for them to do activities independently?		
**Supporting in ADLs or exercise**	The therapist must work with participants to find ways in which the participant can do daily tasks and activities	Does the therapist discuss strategies (e.g. photos, instructions, carer input) with participant to enable them to do their ADLs or exercise? Do they explain how to do them? Do they use a clear language and practical example to support them?		
**Supporting dual-tasking**	The therapist must challenge the participant to complete two exercises at once	Does the therapist ask the participant to do tasks where the mind and the body work at the same time (e.g. walking and counting)? This could be either with the exercise programme or through a functional activity.		
**Accessing the environment**	The therapist must consider ways to maximise physical activity and exercise in the participant's home	Does the therapist ask about, advises on, suggests or gives information on activities that can be done inside the home? Does the therapist discuss full access of the person's property?		
**Embracing positive risk-taking**	Tasks must encourage positive risk-taking and only be discouraged if safety could be compromised	Does the therapist encourage the participant to do tasks where there is a degree of calculated risk? Does the therapist expose the participant to unnecessary risk of harm?<br>Does the therapist discuss positives and negatives of doing more risky activities?<br>Does the therapist use the risk enablement paperwork? Does the therapist consider risk management strategies or contingency plans, when discussing more risky activities?		
**Using Self-Determination Theory principles**	Contact must respond to the human needs for competence (feeling capable of doing the tasks), autonomy (being	Does the therapist give unconditional support and encouragement to boost the participant's confidence? Does the therapist empower the		

For peer review only

	in control of the programme and the tasks)** and relatedness (feeling cared for and connected to the therapist)	participant, by giving them control over the tasks and other aspects of the programme? Is the therapist relatable and working actively to build a human connection with the participant? Does the therapist use the 12 motivational strategies provided by PrAISED team?		
**Assisting in habit formation**	Therapist must assist the participant to develop a habit of being physically active	Does the therapist find ways to integrate the activities into the participant's routine? Do they check that the participant is forming a habit of doing physical activity? Does the therapist use the habit forming strategies provided by PrAISED team?		
**Using tapering to promote self-management**	Therapist must grade the amount of support and supervision provided to participant, to make them more responsible of the activity as time progresses	Does the therapist discuss and agree with participant on the level of intensity of support required to do the tasks and the frequency of next contacts? Is the therapist progressively reducing support (even within the session)? Does the therapist discuss reducing the level of support as programme progresses? Does the therapist use the tapering strategies provided by PrAISED team?		
**Promoting long-term engagement**	The therapist must support the participant to develop intrinsic motivation to ensure that they participants remain active over time	Does the participant seem to enjoy the activity plan? Does it seem that the participants might be able / willing to keep doing the activities over time? Do the therapist work to ensure this, by exploring participant's views?		
**Goal Setting**	The therapist must set goals with the participant that are specific to their interests, functional and active	Does the therapist discuss goal setting with the participant? (sets new goals, review existing goals, adapt/change goals)		

	Does the goal or action plan associated with it lead to the participant doing regular active tasks?		

* Rate as: 1=Visit following principle; 2=visit not following principle; 0=Principle not applicable

For peer review only

BMJ Open

Protocol for the process evaluation of the Promoting Activity, Independence and stability in early Dementia (PrAISED), following changes required by the COVID-19 pandemic

Journal:	*BMJ Open*
Manuscript ID	bmjopen-2020-039305.R2
Article Type:	Protocol
Date Submitted by the Author:	30-Jul-2020
Complete List of Authors:	Di Lorito, Claudio; University of Nottingham,<br>Bosco, Alessandro; University of Nottingham - Jubilee Campus, School of Medicine<br>Goldberg, Sarah; University of Nottingham, School of Health Sciences, University of Nottingham<br>Nair, Roshan<br>O'Brien, Rebecca<br>Howe, Louise<br>van der Wardt, Veronika; Philipps-Universitat Marburg<br>Pollock, Kristian; University of Nottingham, School of Health Sciences<br>Booth, Vicky<br>Logan, Phillipa; Community Health Sciences<br>Godfrey, Maureen<br>Dunlop, Marianne<br>Horne, Jane<br>Harwood, Rowan; University of Nottingham School of Health Sciences; Nottingham University Hospitals NHS Trust, Health Care of Older People
**Primary Subject Heading**:	Sports and exercise medicine
Secondary Subject Heading:	Geriatric medicine, Rehabilitation medicine
Keywords:	GERIATRIC MEDICINE, Dementia < NEUROLOGY, REHABILITATION MEDICINE, SPORTS MEDICINE

SCHOLARONE™ Manuscripts

I, the Submitting Author has the right to grant and does grant on behalf of all authors of the Work (as defined in the below author licence), an exclusive licence and/or a non-exclusive licence for contributions from authors who are: i) UK Crown employees; ii) where BMJ has agreed a CC-BY licence shall apply, and/or iii) in accordance with the terms applicable for US Federal Government officers or employees acting as part of their official duties; on a worldwide, perpetual, irrevocable, royalty-free basis to BMJ Publishing Group Ltd ("BMJ") its licensees and where the relevant Journal is co-owned by BMJ to the co-owners of the Journal, to publish the Work in this journal and any other BMJ products and to exploit all rights, as set out in our licence.

The Submitting Author accepts and understands that any supply made under these terms is made by BMJ to the Submitting Author unless you are acting as an employee on behalf of your employer or a postgraduate student of an affiliated institution which is paying any applicable article publishing charge ("APC") for Open Access articles. Where the Submitting Author wishes to make the Work available on an Open Access basis (and intends to pay the relevant APC), the terms of reuse of such Open Access shall be governed by a Creative Commons licence – details of these licences and which Creative Commons licence will apply to this Work are set out in our licence referred to above.

Other than as permitted in any relevant BMJ Author's Self Archiving Policies, I confirm this Work has not been accepted for publication elsewhere, is not being considered for publication elsewhere and does not duplicate material already published. I confirm all authors consent to publication of this Work and authorise the granting of this licence.

For peer review only

Protocol for the process evaluation of the Promoting Activity, Independence and stability in early Dementia (PrAISED), following changes required by the COVID-19 pandemic

Claudio Di Lorito, Alessandro Bosco, Sarah Goldberg, Roshan das Nair, Rebecca O'Brien, Louise Howe, Veronika van der Wardt, Kristian Pollock, Vicky Booth, Pip Logan, Maureen Godfrey, Marianne Dunlop, Jane Horne, Rowan H. Harwood

Corresponding author: Claudio Di Lorito, email: claudio.dilorito@nottingham.ac.uk

Abstract

Introduction. The PrAISED Randomised Controlled Trial (RCT) is evaluating a home-based, face-to-face, individually tailored, activity and exercise programme for people living with dementia. Social distancing requirements following the COVID-19 pandemic necessitated rapid changes to intervention delivery.

Methods and analysis. A mixed methods process evaluation will investigate how the changes were implemented and the impact that these have on participants' experience. An *implementation study* will investigate how the intervention was delivered during the pandemic. A *study on the mechanisms of impact and context* will investigate how these changes were experienced by the PrAISED participants, their carers and the therapists delivering the intervention. The study will commence in May 2020.

Ethics and dissemination. The PrAISED RCT and process evaluation have received ethical approval number 18/YH/0059. The ISRCTN Registration Number for PrAISED is 15320670. The PrAISED process evaluation will enable us to understand how distancing and isolation affected participants, their activity and exercise routines, and whether the therapy programme could be continued with remote support. This will be valuable both in explaining trial results, and also contribute to understanding and designing new ways of delivering home-based services and rehabilitation interventions for people with dementia and their carers.

Strengths and limitations of this study

- This study will capture the full range of perspectives, by involving in research participants with dementia, their carers and professionals delivering the intervention;
- This study will gather a holistic picture of the phenomenon, as it uses different methodologies, including quantitative and qualitative data, and data triangulation;

For peer review only - http://bmjopen.bmj.com/site/about/guidelines.xhtml

- This study will collect qualitative data at two time points, to capture progress over time;
- The qualitative interviews in this study will be carried out remotely, which could pose barriers to participants with dementia;
- This process evaluation team is not independent of the main trial team and this may generate confirmation bias of study hypotheses

For peer review only

Introduction

Dementia is a neurodegenerative condition characterised by a cluster of symptoms, including memory loss and deterioration of motor skills (1-4). More than 50 million people in the world live with dementia (5). Projections estimate that this number will rise to 130 million people in the next 30 years (5). Dementia presents enormous financial burden (6). In the United Kingdom alone, the cost of health and social care for people with the condition is £50 billion, which will grow to £140 billion by 2040 (5). Keeping physically active has benefits for people with dementia on executive functioning, mobility, activities of daily living, independence, and quality of life (7-25), which have been linked to reduced risk of falls, hospital admissions and health and social care costs.

A number of physical activity and exercise intervention programmes have been developed for people with dementia (15,16). Among these is the Promoting Activity, Independence and Stability in Early Dementia (PrAISED) (26), an intervention to promote activity and independence in people with early dementia or mild cognitive impairment, whose clinical and cost-effectiveness is being evaluated in a five-site Randomised controlled Trial (RCT). So far, out of a total recruitment target of 368 participants, 300 participants have been randomised to either a control group (brief falls assessment and advice) or an intervention arm (27). Participants in the intervention arm receive an individually-tailored programme of up to 50 visits at home over a period of 52 weeks from a multidisciplinary team including physiotherapists (PTs), occupational therapists (OTs) and rehabilitation support workers (RSWs) (27). The PrAISED programme comprises: physical exercises (i.e. progressive strength, balance and dual-task); functional activities (i.e. activities of daily living with an element of physical activity, such as going out for food shopping); promotion of inclusion in community life (e.g. through provision of information on physical exercise group classes); risk enablement (assessing, mitigating and agreeing on risks to be taken or avoided); and environmental assessment (27).

The PrAISED RCT includes a process evaluation (28), which aims to describe and quantify intervention delivery, identify the key elements that make the intervention effective and the variables affecting participants motivation to adhere to the programme and remain physically active in the long-term (i.e. beyond the active intervention period). These variables, which have been recently synthesised in a theoretical model (29,30), include the social opportunities linked to exercise, the therapeutic relationship built up with the therapists delivering the intervention, family or carer support, the availability and inclusion of the person in community (physical) activities, the accessibility of the environment (e.g. availability of parks, public transport) and the notion of independence and autonomy (e.g. how, when, and where to exercise).

In March 2020, many of the elements enabling and supporting participants in the PrAISED programme became impossible to deliver due to the pandemic of COVID-19. Measures to slow the spread of the virus were advised and then mandated by governments (31-33). People over 70 years of age, especially those with pre-existing conditions, were told to self-isolate to shield them from increased risk of illness, complications, hospitalisation and mortality (34, 35).

For peer review only - http://bmjopen.bmj.com/site/about/guidelines.xhtml

The negative effects that social isolation may have on the health and well-being of older people are well known (36). In people with dementia, there might be additional effects, such as a negative impact on functioning, through loss of opportunity to engage with family or in activities outside the home. In order to continue the trial and maintain an element of social contact during this unprecedented time, changes were made to the PrAISED programme intervention delivery (Table 1). There were no changes in training, as all therapists delivering PrAISED were recruited and trained before the amendment to PrAISED. Instead, the therapists were provided with new written guidance on how to deliver the intervention remotely (Appendix 1). The participants who were still receiving the intervention when these changes occurred (March 2020) (n=213) automatically started receiving the amended version of the PrAISED programme. The main change was that participants would not receive visit from therapists at home, as this would place them at risk of contracting the virus. Instead, therapists would continue to support the participants remotely, by telephone or video, in line with the Chartered Society of Physiotherapists guidance (37).

These changes might have important implications on the participants' experience of the intervention. Previous studies have found that face-to-face support from therapists facilitates the creation of a strong therapeutic alliance with the person with dementia, which proves an effective tool for adherence (30). Home visits may facilitate co-production of a programme tailored to the person's needs and aspirations, which is linked to feelings of empowerment and autonomy (38). They may also prove positive for the carers, who, as a result of their caring duties, may risk social isolation (39,40) and reduced quality of life (41). On the other hand, face-to-face support can increase feelings of dependency among participants, potentially resulting in separation anxiety toward the end of the intervention period (30). From the therapists' perspective, delivering an intervention in the participants' homes can be time consuming. It has been reported in previous process evaluations that adding travelling times on top of the existing workload might thwart job satisfaction (42). The use of remote support might rectify some of these negative experiences.

We aim to extend the process evaluation of the PrAISED (28), to investigate the impact of the changes made to PrAISED. Specifically, the proposed study will respond to the research questions:

- How does staying at home, with no current possibility of receiving face-to-face support from therapists, affect the uptake and retention of a physical activity and exercise programme in participants with dementia? How does it affect their ability to remain independent and their quality of life? Are there ways in which people with dementia can be better supported to remain physically active and independent in these circumstances?
- How are therapists supported to deliver a physical activity and exercise programme remotely to participants with dementia? How does this support affect their confidence and ability to deliver the intervention? Are there ways in which therapists can better supported to deliver the intervention remotely?

Methods and analysis

For peer review only - http://bmjopen.bmj.com/site/about/guidelines.xhtml

Based on the assumption that *'if intervention X (i.e. PrAISED) is delivered, the mediating variable(s) (e.g. staying at home, support from therapists available only remotely) affects the way in which outcome Y (e.g. uptake and retention of a physical activity and exercise) will occur'*, a process evaluation aims to understand how an intervention works (43). It does so by studying the 'implementation of the intervention' (e.g. how the intervention is delivered), the 'mechanisms of impact' (e.g. how participants respond individually to the intervention being delivered) and the 'context' (e.g. the physical and social environment affecting participants' response to the intervention) (43).

This process evaluation will adopt a mixed-methods approach, including quantitative data and data ensuing from qualitative interviews. It will consist of two studies: an implementation study and a study on mechanisms of impact and context (Figure 1). The study will commence in May 2020 and the final results are expected to be available in May 2021.

Patient and Public Involvement

The process evaluation study team includes two patient and public involvement (PPI) contributors (MG and MD), who have been involved in the development of the process evaluation and its protocol (also acting as co-authors). The PPI contributors co-designed with the main researcher (CDL) the topic guide for the qualitative interviews of participants with dementia and their carers (see details in "study of mechanisms of impact and context – data collection") and will be involved as co-raters in the qualitative analysis of the transcripts of the interviews (see details in "study of mechanisms of impact and context – data analysis") and in disseminating research findings (e.g. through attending conferences, public dissemination events and co-authoring results' papers).

Implementation study

The study on implementation will investigate how the PrAISED intervention is delivered, following changes in procedure in response to the COVID-19 pandemic. It will focus on four domains (Table 2):

- Fidelity (i.e. the consistency of delivery of PrAISED with the amended protocol);
- Adaptations (i.e. alterations made to delivery of PrAISED to achieve better contextual fit);
- Dose (i.e. how much PrAISED intervention is delivered);
- Reach (i.e. the number of therapists trained to deliver PrAISED and of participants who receive the intervention).

Participants

The implementation study will include participants with dementia in the intervention group, their carers and therapists who are involved in the PrAISED main trial at the time of recruitment (May 2020).

Data collection

From the participants with dementia:

- Adherence to intervention as per instructions (Fidelity), investigated through qualitative interviewing;
- Adherence to advised activity levels (Dose), investigated through minutes of PrAISED activity per week as recorded on a self- (or carer-) completed monthly calendar;
- The extent to which participants with dementia come into contact with the intervention (Reach), investigated by totalling the number of participants who completed the programme;
- Alterations that participants made to achieve better contextual fit (Adaptations), investigated through qualitative interviewing.

From the therapists:

Evaluation of the delivery of the adapted intervention, including:

- Number and length of remote sessions the therapists have with participants (dose and reach): A record of the date, length in minutes, and therapist type (PT, OT and RSW) will be recorded for each contact. The information is collated by the research team each week.
- Goals set for participants (adaptations): Goals that have been set with the participants are documented by the therapists and collated centrally by the research team.
- Intervention content (fidelity, adaptations): One intervention session provided remotely by each therapist will be audio-recorded. To ensure safe handling and storing of sensitive data, the session between the therapist and the participant will be recorded remotely by one researcher within the PrAISED team with an encrypted digital audio recorder.

Data analysis

The data from the implementation study will be analysed using IBM SPSS Statistics version 26 (44). Descriptive statistical analysis will be used to measure fidelity, dose and reach.

The audio recordings will be transferred onto an encrypted and password protected university computer server. The content will be assessed independently by two raters against 14 core principles set out in the PrAISED therapists' training manual (i.e. *'visit following core principle', 'visit not following core principle', 'Principle not applicable'*). An audio-analysis

For peer review only - http://bmjopen.bmj.com/site/about/guidelines.xhtml

template will list the core principles, provide operational definitions of each of them, accompanied with practical examples of the application of principle, to facilitate retrieval of content during analysis (Appendix 2).

Prior to independent audio analysis, the two raters will pilot-test the rating procedure using a sample audio recording, to check inter-rater reliability. Scores from the two raters will be compared to determine inter-rater reliability, and if inconsistency arises in scoring, consensus will be reached through discussion between the two raters or through involvement of a third rater.

Study on mechanisms of impact and context

The study on mechanisms of impact and context will investigate the participants and therapists' experience of the intervention, and any variable mediating intervention outcomes (e.g. social distancing).

Participants

For each research site, we will include:

1. Participants with dementia and their carer, further divided in:

- Intervention arm (i.e. receiving the active intervention);
- Control arm (i.e. receiving treatment as usual, included to investigate whether there are any relevant differences between control and intervention arm);
- Those who have withdrawn from the therapy programme, if they agree to be interviewed.

Purposive sampling will be carried out to ensure a diverse and representative sample in relation to gender, ethnicity, residence status (i.e. living independently or living with carer) and the different research sites involved in PrAISED (i.e. Nottinghamshire, Derbyshire, Lincolnshire, Somerset and Oxfordshire). The main researcher (CDL) will access the PrAISED RCT database and select participants from the different subgroups.

We will not exclude participants who do not have mental capacity to agree to participate or who show fluctuating capacity at the point of the interview, for the following reasons: Firstly, they might still provide precious insight into the mechanisms of the intervention; secondly, their (fluctuating) cognition may have an impact and affects their response toward the intervention; finally, from an ethical standpoint, we aim to give voice to all those whose life is primarily affected by our research. However, we will take into account capacity to give consent (or lack thereof) during the course of the interview, by relying, for example, on different degrees of carer support during the session.

2. Therapists will be purposively sampled to be involved in the process evaluation. The main researcher (CDL) will access the PrAISED RCT database and select therapists

For peer review only - http://bmjopen.bmj.com/site/about/guidelines.xhtml

from the different professions (i.e. physiotherapists, occupational therapists and rehabilitation support workers) and research sites.

In line with Guest, Bunce and Johnson (45), we argue that, given the lack of guidance around reaching data saturation, there is a need to adopt appropriate 'tests of adequacy' for sample sizes in qualitative research. Based on the notion of 'conceptual density' (i.e. gathering data until a *sufficient depth* of understanding of the domains under investigation is reached) (46), we will adopt a *Conceptual Depth Scale* developed by Nelson (46) (Table 3), which assigns a score ranging from 1 (low) to 3 (high) to establish whether conceptual density is reached in relation to:

- *'Range'* (e.g. extent of diversity of data sources);
- '*Complexity'* (e.g. extent of networks / links across data);
- '*Subtlety'* (e.g. extent of similarity across data);
- *'Validity'* (e.g. extent to which data are transferable to other settings)

The scoring will be performed by two researchers independently of each other. The scale is used as instrument to check whether consensus is reached among researchers with respect to data saturation, rather than as quantitative assessment to determine a saturation point for data interpretation. We anticipate that conceptual density will be reached by inclusion of up to 20 participants with dementia (and 20 carers), and 20 therapists across all research sites.

Data collection

The investigation of the mechanisms of impact and context will be based on qualitative interviews with participants. The first interview will be conducted one month following the change of intervention in response to the COVID-19 pandemic (i.e. May 2020). Follow up interviews will be considered, if the measures imposed following the COVID-19 pandemic are still in place, to monitor progress over time.

The interviews will consist of:

- Remote interviews (different options will be offered, including telephone or video calling, depending on participants' preference) with participants with dementia and their carers (as a dyad, so that the carer can provide information, as well as support, if needed). We will use a speaker phone for everyone to be able to contribute. Prior to the session, the researcher will mail (or email) a copy of the consent form. A verbal consent for both the participant with dementia and the carer will be recorded on tape, before the interview begins.

- Remote interviews with therapists (i.e. occupational therapist, physiotherapist and rehabilitation support worker). Verbal consent will be recorded on tape prior to the interview.

For peer review only - http://bmjopen.bmj.com/site/about/guidelines.xhtml

The topic guide for the qualitative interviews is informed by the *PHYT in dementia* (PHYsical activity behaviour change Theory in dementia), whose development and validation we reported elsewhere (29,30). Through this theoretical framework, we identified potential variables mediating intervention outcomes and developed several prompts to stimulate discussion. Exploration of context will include the impact of isolation, and its effects on exercise, activity and mental well-being.

We developed the topic guides as a collaborative effort between the research team and the PPI contributors, who helped to ensure that the interview prompts are relevant, meaningful and accessible for the participants. Although questions are study-specific, the prompts are broad in scope, to ensure that the participants feel free to express their ideas around unanticipated causal processes and consequences. The participants may also raise additional topics and issues which they feel are particularly relevant in the context of the COVID-19 pandemic, and these will be explored accordingly.

The qualitative interviews are expected to last around 40 minutes, depending on participants' engagement in the process, their cognitive abilities, and logistics.

Data analysis

Data will be analysed through framework analysis (47). This method is ideal in social and health care qualitative research studies with large data sets. Framework analysis will ensure in-depth exploration of data, a transparent audit trail of the process of analysis, and the understanding of data interpretation (e.g. a description of how data link to each other and according to the objective of the study) through visual mapping (47).

Data analysis will follow the steps for good practice in Framework Analysis identified by Gale et al. (47):

1. *Verbatim transcription* of the interviews by a professional transcriber, who will also anonymise data. Large margins and double line spacing in the transcripts will be left to create room for coding and note taking.
2. *Familiarisation with the transcripts* by the main researcher (CDL), who will write down analytical notes on margins.
3. *Coding of a sample of three transcripts* by the main researcher, a second researcher within the research team and one PPI contributor, who will independently underline relevant pieces of text and write coding labels for each, reflecting the constructs included in the topic guide. However, to prevent the omission of important data, if novel constructs are identified from the transcripts, new coding labels will be generated.
4. *Development of a working analytical framework* through team work of the three coders, who will create a set of initial codes through synthesis of individual coding and operational definitions. Two more transcripts will be coded by two coders to check whether the initial working analytical framework is suitable. Eventually, a stable set of codes, clustered into umbrella categories will be identified.

For peer review only - http://bmjopen.bmj.com/site/about/guidelines.xhtml

5. *Use of the working analytical framework* by the main researcher (CDL) to code the whole set of transcripts in NVivo 12 (48). Double coding will be conducted by another researcher.
6. *Charting of data into the framework matrix* by the main researcher on NVivo. The matrix will map out codes (one per column) and participants (one per row). The relevant quotes will be transferred from NVivo onto the matrix.
7. *Interpretation of data* by the main researcher, who will develop themes from the matrix by making connections within and between participants and categories. This will be an iterative process, with regular review from members of the research team.

Ethics and dissemination

The PrAISED trial and process evaluation have received ethical approval number 18/YH/0059. The ISRCTN Registration Number for PrAISED is 15320670.

This protocol, grounded in the MRC framework for process evaluation of complex intervention (43), outlines the rationale, design and methods for the process evaluation of the Promoting Activity, Independence and Stability in Early Dementia and mild cognitive impairment (PrAISED), following the changes made as a result of the restrictions on face-to-face contact during the COVID-19 pandemic.

In only a few months, the COVID-19 pandemic has required dramatic changes to our lifestyles and caused unprecedented operational strain on national health and social care systems. There is a need for scientific evidence to inform research and services in response to the current challenges, as well as preparation for services after the pandemic and potential future events. In these respects, the final process evaluation report, which will be disseminated in scientific journals and to the public (e.g. through public engagement events), will report on the impact that the social distancing measures introduced in PrAISED have had on research participants and therapists. By comparing the evidence gathered through this study with the original PrAISED process evaluation (28) and the wider literature, this process evaluation will contribute knowledge on ways in which individuals belonging to the most vulnerable groups in society can be better supported and motivated to remain physically active and healthy in their homes without face-to-face support. In addition, by triangulating data from this process evaluation with some quantitative measures from the RCT (e.g. quality of life (QoL) and carer strain), we will be able to gather a more comprehensive picture of the impact that the COVID-19 has had on the lives of participants.

This work will also present important implications in theory advancement. Our dissemination plans include a paper further validating the *PHYT in dementia*, the behaviour change theoretical model that our research team previously developed and validated through data from the original PrAISED process evaluation (29,30). Results from this work will contribute further evidence to confirm / challenge the validity of the model in explaining motivation to be physically active, in the context of social distancing. Finally, based on findings from this process evaluation, we aim to develop a methodological paper outlining strategies that can be used to involve research participants remotely in an ethical, meaningful and practically feasible way. This model can be refined through input from research teams conducting

For peer review only - http://bmjopen.bmj.com/site/about/guidelines.xhtml

rehabilitation studies in similar circumstances, such as the FinCH study (49), to derive a research platform that can be shared to inform / guide good practice in future research.

In conclusion, this process evaluation represents one of the first efforts to document how an ongoing research programme was adapted as a result of the COVID-19 pandemic. This study will support the critical reflection by the PrAISED team on positive and negative aspects of these adaptations. It will also provide transferable information to develop strategies to effectively deliver rehabilitation remotely, in the presence of extraordinary circumstances (e.g. social distancing and staying at home).

Funding statement: This protocol presents independent research funded by the United Kingdom National Institute for Health Research (NIHR) under its Programme Grants for Applied Research funding scheme (RP-PG-0614-20007). The views expressed are those of the authors and not necessarily those of the National Health Service, the NIHR or the Department of Health and Social Care.

Conflict of interest: None to declare

Authors' contributions: CDL contributed to the conception and design of the study, and the development of all the elements of the process evaluation. AB contributed to the planning of the process evaluation, the analysis plan for therapists' audio recordings, and provided feedback and final approval of the manuscript. SG contributed the PrAISED RCT information, helped in the conception of the study, and provided feedback and final approval of the manuscript. RdN contributed to the development of the analysis plan for the qualitative element of the study and the therapists' audio recordings, and provided feedback and final approval of the manuscript. ROB contributed to develop the study design and the analysis plan of the therapists' audio recordings, and provided feedback and final approval of the manuscript. LH helped to develop the topic guide for the therapists' qualitative interviews, as well as the analysis plan, and provided feedback and final approval of the manuscript. VvdW contributed to the study conception and design, the implementation study and the quantitative data analysis plan, and provided feedback and final approval of the manuscript. KP provided guidance and expertise on the development of the qualitative interviews for participants with dementia and carers, and provided feedback and final approval of the manuscript. VB contributed to the design and analysis plan of the implementation study, and provided feedback and final approval of the manuscript. PL contributed to the discussion section of the manuscript, and provided feedback and final approval of the manuscript. MG and MD were the PPI collaborators of the study. They contributed to the development of the topic guide for the qualitative interviews of the participants with dementia and their carers, and provided feedback and final approval of the manuscript. JH contributed to the discussion section of the manuscript, and provided feedback and final approval of the manuscript. RHH contributed the PrAISED RCT information, helped in the conception of the study and provided feedback and final approval of the manuscript.

Words: 3525

For peer review only - http://bmjopen.bmj.com/site/about/guidelines.xhtml

Figure 1. Method of Process Evaluation

Reference

(1). Martyr, A. and L. Clare, Executive function and activities of daily living in Alzheimer's disease: a correlational meta-analysis. Dementia and geriatric cognitive disorders, 2012. 33(2-3): p. 189-203.

(2). Giebel, C.M., et al., Deterioration of basic activities of daily living and their impact on quality of life across different cognitive stages of dementia: a European study. International Psychogeriatrics, 2014. 26(8): p. 1283-1293.

(3). Giebel, C.M., C. Sutcliffe, and D. Challis, Activities of daily living and quality of life across different stages of dementia: a UK study. Aging & Mental Health, 2015. 19(1): p. 63-71.

(4). Alzheimer's Research UK (2019). About dementia. Available online: https://www.alzheimersresearchuk.org/about-dementia/?gclid=Cj0KCQjw3JXtBRC8ARIsAEBHg4myuYeahFMLCcSGLr-flQxznmdB-dObW2gXc5MUN9o_dfNw5wwI5EwaAvH6EALw_wcB (accessed on 26 March 2020).

(5). Alzheimer's Society (2020). Facts for the Media. Available online: https://www.alzheimers.org.uk/about-us/news-and-media/facts-media (accessed on 26 March 2020).

(6). Lewis F, Karlsberg Schaffer S, Sussex J, O'Neill P, Cockcroft L. The trajectory of dementia in the UK-making a difference. Office of Health Economics Consulting Reports. 2014 Jun 1.

(7). Forbes D, Forbes SC, Blake CM, Thiessen EJ, Forbes S. Exercise programs for people with dementia. Cochrane Database of Systematic Reviews. 2015(4). doi:10.1002/14651858.CD006489.pub4.

(8). Potter R, Ellard D, Rees K, Thorogood M. A systematic review of the effects of physical activity on physical functioning, quality of life and depression in older people with dementia. International journal of geriatric psychiatry. 2011 Oct;26(10):1000-11.

(9). Blankevoort CG, Van Heuvelen MJ, Boersma F, Luning H, De Jong J, Scherder EJ. Review of effects of physical activity on strength, balance, mobility and ADL performance in elderly subjects with dementia. Dementia and geriatric cognitive disorders. 2010;30(5):392-402.

(10). Heyn P, Abreu BC, Ottenbacher KJ. The effects of exercise training on elderly persons with cognitive impairment and dementia: a meta-analysis. Archives of physical medicine and rehabilitation. 2004 Oct 1;85(10):1694-704.

For peer review only

(11). Hauer K, Schwenk M, Zieschang T, Essig M, Becker C, Oster P. Physical training improves motor performance in people with dementia: a randomized controlled trial. Journal of the American Geriatrics Society. 2012 Jan;60(1):8-15.

(12). Hoffmann K, Sobol NA, Frederiksen KS, Beyer N, Vogel A, Vestergaard K, Brændgaard H, Gottrup H, Lolk A, Wermuth L, Jacobsen S. Moderate-to-high intensity physical exercise in patients with Alzheimer's disease: a randomized controlled trial. Journal of Alzheimer's Disease. 2016 Jan 1;50(2):443-53.

(13). Lamb SE, Sheehan B, Atherton N, Nichols V, Collins H, Mistry D, Dosanjh S, Slowther AM, Khan I, Petrou S, Lall R. Dementia And Physical Activity (DAPA) trial of moderate to high intensity exercise training for people with dementia: randomised controlled trial. bmj. 2018 May 16;361:k1675.

(14). Lowery, D., Cerga-Pashoja, A., Iliffe, S., Thuné-Boyle, I., Griffin, M., Lee, J., Bailey, A., Bhattacharya, R. and Warner, J., 2014. The effect of exercise on behavioural and psychological symptoms of dementia: the EVIDEM-E randomised controlled clinical trial. *International journal of geriatric psychiatry*, *29*(8), pp.819-827.

(15). Pitkälä KH, Pöysti MM, Laakkonen ML, Tilvis RS, Savikko N, Kautiainen H, Strandberg TE. Effects of the Finnish Alzheimer disease exercise trial (FINALEX): a randomized controlled trial. JAMA internal medicine. 2013 May 27;173(10):894-901.

(16). Prick AE, De Lange J, Scherder E, Twisk J, Pot AM. The effects of a multicomponent dyadic intervention with physical exercise on the cognitive functioning of people with dementia: A randomized controlled trial. Journal of aging and physical activity. 2017 Oct 1;25(4):539-52.

(17). Rolland Y, Pillard F, Klapouszczak A, Reynish E, Thomas D, Andrieu S, Rivière D, Vellas B. Exercise program for nursing home residents with Alzheimer's disease: a 1-year randomized, controlled trial. Journal of the American Geriatrics Society. 2007 Feb;55(2):158-65.

(18). Schwenk M, Zieschang T, Oster P, Hauer K. Dual-task performances can be improved in patients with dementia: a randomized controlled trial. Neurology. 2010 Jun 15;74(24):1961-8.

(19) Schwenk M, Zieschang T, Englert S, Grewal G, Najafi B, Hauer K. Improvements in gait characteristics after intensive resistance and functional training in people with dementia: a randomised controlled trial. BMC geriatrics. 2014 Dec;14(1):73.

(20). Suzuki T, Shimada H, Makizako H, Doi T, Yoshida D, Tsutsumimoto K, Anan Y, Uemura K, Lee S, Park H. Effects of multicomponent exercise on cognitive function in older adults with amnestic mild cognitive impairment: a randomized controlled trial. BMC neurology. 2012 Dec 1;12(1):128.

(21). Telenius EW, Engedal K, Bergland A. Effect of a high-intensity exercise program on physical function and mental health in nursing home residents with dementia: an assessor blinded randomized controlled trial. PloS one. 2015;10(5).

(22). Forbes D, Forbes SC, Blake CM, Thiessen EJ, Forbes S. Exercise programs for people with dementia. Cochrane Database of Systematic Reviews. 2015(4).

For peer review only - http://bmjopen.bmj.com/site/about/guidelines.xhtml

(23). Potter R, Ellard D, Rees K, Thorogood M. A systematic review of the effects of physical activity on physical functioning, quality of life and depression in older people with dementia. International journal of geriatric psychiatry. 2011 Oct;26(10):1000-11.

(24). Blankevoort CG, Van Heuvelen MJ, Boersma F, Luning H, De Jong J, Scherder EJ. Review of effects of physical activity on strength, balance, mobility and ADL performance in elderly subjects with dementia. Dementia and geriatric cognitive disorders. 2010;30(5):392-402.

(25). Heyn P, Abreu BC, Ottenbacher KJ. The effects of exercise training on elderly persons with cognitive impairment and dementia: a meta-analysis. Archives of physical medicine and rehabilitation. 2004 Oct 1;85(10):1694-704.

(26). Booth V, Harwood RH, Hood-Moore V, Bramley T, Hancox JE, Robertson K, Hall J, Van Der Wardt V, Logan P. Promoting Activity, Independence and Stability in Early Dementia and Mild Cognitive Impairment (PrAISED): Development of an Intervention for People with Mild Cognitive Impairment and Dementia. Clinical Rehabilitation 2018; 32: 855-864. doi.org/10.1177/0269215518758149

(27). Bajwa RK, Goldberg SE, Van der Wardt V, Burgon C, Di Lorito C, Godfrey M, Dunlop M, Logan P, Masud T, Gladman J, Smith H. A randomised controlled trial of an exercise intervention promoting activity, independence and stability in older adults with mild cognitive impairment and early dementia (PrAISED)-A Protocol. Trials. 2019 Dec;20(1):1-1.

(28). Di Lorito C, Pollock K, Harwood R, das Nair R, Logan P, Goldberg S, Booth V, Godfrey M, Dunlop M, Van der Wardt V. Protocol for the process evaluation of the promoting activity, independence and stability in early dementia and mild cognitive impairment (PrAISED 2) randomised controlled trial. Maturitas. 2019 Apr 1;122:8-21.

(29). Di Lorito C, Pollock K, Harwood R, das Nair R, Logan P, Goldberg S, Booth V, Vedhara K, Van Der Wardt V. A scoping review of behaviour change theories in adults without dementia to adapt and develop the 'PHYT in dementia', a model promoting physical activity in people with dementia. Maturitas. 2019 Mar 1;121:101-13.

(30). Di Lorito C, Bosco A, Pollock K, H Harwood R, Das Nair R, Logan P, Goldberg S, Booth V, Vedhara K, Godfrey M, Dunlop M. External Validation of the 'PHYT in Dementia', a Theoretical Model Promoting Physical Activity in People with Dementia. International Journal of Environmental Research and Public Health. 2020 Jan;17(5):1544.

(31). World Health Organization (2020). WHO Director-General's opening remarks at the media briefing on COVID-19 - 11 March 2020. Retrieved from: https://www.who.int/dg/speeches/detail/who-director-general-s-opening-remarks-at-the-media-briefing-on-covid-19---11-march-2020 (accessed: 02 April 2020).

(32). Ferguson NM, Laydon D, Nedjati-Gilani G, Imai N, Ainslie K, Baguelin M, Bhatia S, Boonyasiri A, Cucunubá Z, Cuomo-Dannenburg G, Dighe A. Impact of Non-Pharmaceutical Interventions (NPIs) to Reduce COVID-19 Mortality and Healthcare Demand (16 March 2020). URL https://doi. org/10.25561/77482.

(33). Sun P, Lu X, Xu C, Sun W, Pan B. Understanding of COVID-19 based on current evidence. Journal of medical virology. 2020 Feb 25.

For peer review only - http://bmjopen.bmj.com/site/about/guidelines.xhtml

(34). Public Health England (2020). Guidance on social distancing for everyone in the UK. Available at: https://www.gov.uk/government/publications/covid-19-guidance-on-social-distancing-and-for-vulnerable-people/guidance-on-social-distancing-for-everyone-in-the-uk-and-protecting-older-people-and-vulnerable-adults (Accessed: 24 March 2020)

(35). Zu ZY, Jiang MD, Xu PP, Chen W, Ni QQ, Lu GM, Zhang LJ. Coronavirus disease 2019 (COVID-19): a perspective from China. Radiology. 2020 Feb 21:200490.

(36). Hawton A, Green C, Dickens AP, Richards SH, Taylor RS, Edwards R, Greaves CJ, Campbell JL. The impact of social isolation on the health status and health-related quality of life of older people. Quality of Life Research. 2011 Feb 1;20(1):57-67.

(37) The Chartered Society of Physiotherapy. COVID-19: guide for rapid implementation of remote consultations. Retrieved from: https://www.csp.org.uk/publications/covid-19-guide-rapid-implementation-remote-consultations Accessed on 09 April 2020.

(38). Bosco A, Schneider J, Coleston-Shields DM, Orrell M. Dementia care model: Promoting personhood through co-production. Archives of gerontology and geriatrics. 2019 Mar 1;81:59-73.

(39). Brodaty H, Hadzi-Pavlovic D. Psychosocial effects on carers of living with persons with dementia. Australian & New Zealand Journal of Psychiatry. 1990 Sep;24(3):351-61.

(40). Graham C, Ballard C, Sham P. Carers' knowledge of dementia, their coping strategies and morbidity. International journal of geriatric psychiatry. 1997 Sep;12(9):931-6.

(41). Gaugler JE, Kane RL, Kane RA, Newcomer R. Unmet care needs and key outcomes in dementia. Journal of the American Geriatrics Society. 2005 Dec;53(12):2098-105.

(42). Bosco A, Paulauskaite L, Hall I, Crabtree J, Soni S, Biswas A, Cooper V, Poppe M, King M, Strydom A, Crawford MJ. Process evaluation of a randomised controlled trial of PBS-based staff training for challenging behaviour in adults with intellectual disability. PloS one. 2019;14(8).

(43). Moore GF, Audrey S, Barker M, Bond L, Bonell C, Hardeman W, Moore L, O'Cathain A, Tinati T, Wight D, Baird J. Process evaluation of complex interventions: Medical Research Council guidance. bmj. 2015 Mar 19;350.

(44). IBM Corp. Released 2017. IBM SPSS Statistics for Windows, Version 25.0. Armonk, NY: IBM Corp.

(45). Guest G, Bunce A, Johnson L. How many interviews are enough? An experiment with data saturation and variability. Field methods. 2006 Feb;18(1):59-82.

(46). Nelson J. Using conceptual depth criteria: addressing the challenge of reaching saturation in qualitative research. Qualitative research. 2017 Oct;17(5):554-70.

(47). Gale NK, Heath G, Cameron E, Rashid S, Redwood S. Using the framework method for the analysis of qualitative data in multi-disciplinary health research. BMC medical research methodology. 2013 Dec;13(1):117.

(48). NVivo qualitative data analysis Software (2018). Melbourne, Australia: QSR International Pty Ltd.

For peer review only - http://bmjopen.bmj.com/site/about/guidelines.xhtml

(49). Logan P, McCartney K, Armstrong S, Clarke A, Conroy S, Darby J….& Horne J. Evaluation of the Guide to Action Care Home fall prevention programme in care homes for older people: protocol for a multi-centre, single blinded, cluster randomised controlled trial (FinCH). East Midlands Research into Ageing Network (EMRAN) Discussion Paper Series Issue 25 Feb 2019. ISSN 2059-3341.

For peer review only

Table 1. Main changes made to the PrAISED intervention, compared to the original version (26,27)

Delivery of intervention	Provision of support to the therapists
The therapists were provided with written guidance on how to deliver the intervention (Appendix 1)	Increased access to:<br>• Monthly teleconferences across all sites;<br>• Teleconferences at individual sites;<br>• Provision of a regularly updated list of resources;<br>• Provision of informal support through email and phone;<br>• Provision of information and support tailored to the situation and change in practice

For peer review only - http://bmjopen.bmj.com/site/about/guidelines.xhtml

Table 2. Implementation study

	Delivery (Therapists)	*Adherence (Participants)*
Fidelity	Delivery of intervention against PrAISED 2 principles (through audio content)	Adherence to intervention as per instructions (through interview)
Dose	Frequency and length of contact sessions with participant*	Minutes per week recorded on calendar*
Reach	Number of contact sessions with participant*	Number of participants who completed the programme*
Adaptations	Adaptations made to deliver the sessions (through interview)	Adaptations that participants made to physical activity and exercise (through interview)

* Data gathered during main Trial

For peer review only - http://bmjopen.bmj.com/site/about/guidelines.xhtml

Table 3. Conceptual Depth Scale (46)

**Criteria (with sources of evidence)**	**Low (1)**	**Medium (2)**	**High (3)**
Range (e.g. frequency and variety of codes; multiplicity of data sources)	Few examples to sup-port concepts. Only a single data-type	→	Abundant examples to sup-port concepts. Multiple data-types
Complexity (e.g. coding trees; positional maps; matrices)	Descriptive codes; simple or basic connections between codes; low level analysis	→	Sophisticated networks; abstract conceptual categories which synthesise a range of codes and concepts
Subtlety (e.g. memos; social worlds diagrams)	Conceptual language is regarded as unproblematic and one dimensional	→	Conceptual language is understood as rich, ambiguous and multi-dimensional
Resonance (literature)	Weak resonance; emerging theory is re-mote from existing literature and theoretical frameworks	→	Strong resonance; emerging theory makes sense along-side existing literature; there are correlations with other theoretical frameworks, albeit with variations and novel-ties
Validity (e.g. applicability test)	Low level theorising and inward facing; the findings have limited application to the re-search participants or those familiar with similar contexts.	→	Abstract level theorising and outward facing; the findings make sense to those in the social context of the re-search, or ones broadly similar.

For peer review only

Figure 1. Method of Process Evaluation

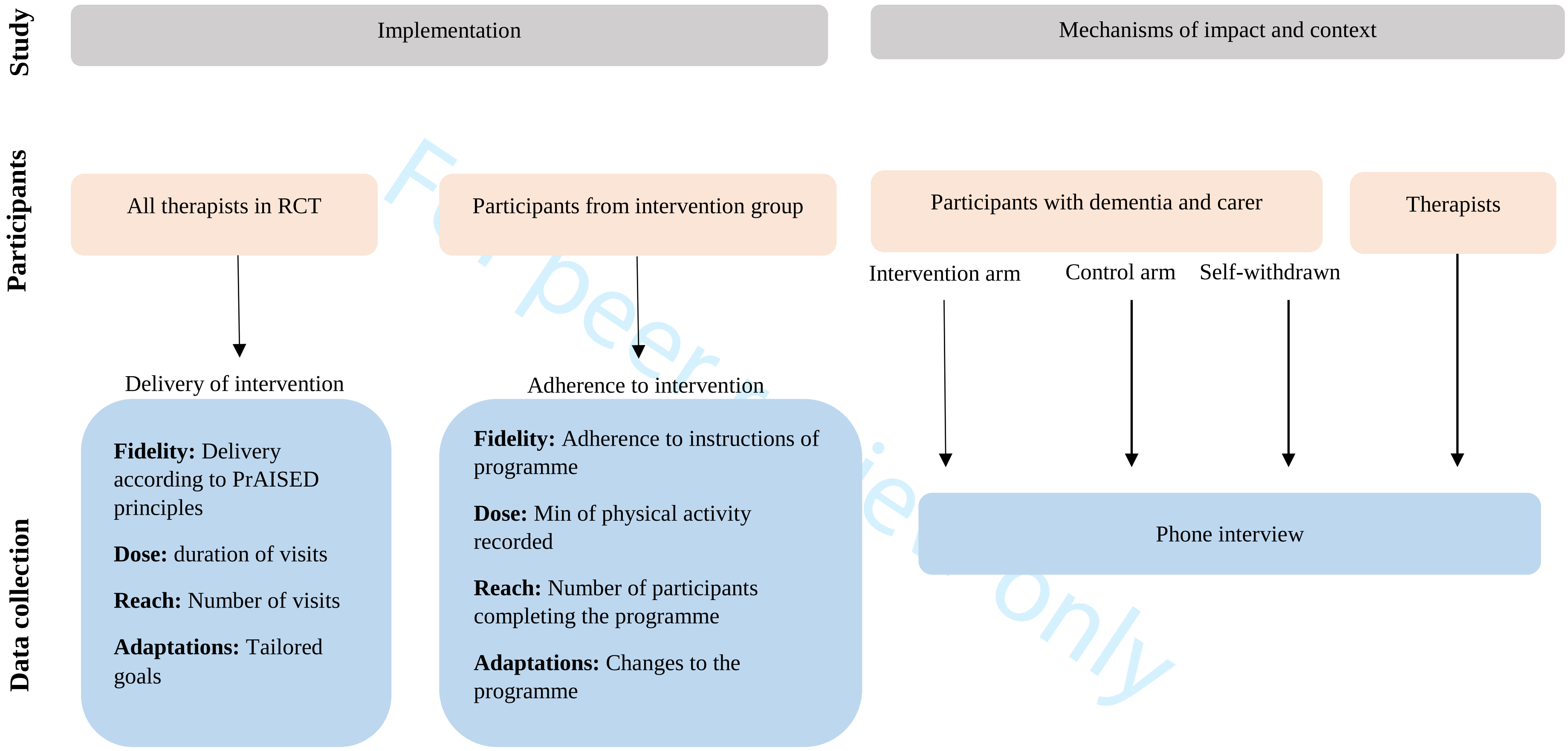

For peer review only

Appendix 1. Guidance distributed among the PrAISED therapists on the changes made to the intervention

Plan for PrAISED2 Intervention in response to COVID19 restrictions (18.03.2020)

Immediate plan

The NIHR have stated that their funded studies should stop all non-essential face to face contact. The PrAISED intervention is not considered essential care and therefore we must stop all face to face contact with our participants.

However, because we have a duty of care to our patients considering many of them will be following the governments advice to reduce all social contact, we have devised a contingency plan to continue with the PrAISED intervention.

Intervention Group Participants

Therapy teams should contact all participants currently in the trial, or their carers if more appropriate, to explain the change in practice as below.

On-going Intervention Group Participants

Visits to participants should be replaced with **telephone coaching** as per their normal schedule, in terms of frequency. For example, if you are seeing someone weekly, this should be continued until they reach the time to reduce to fortnightly. This is the example frequency schedule set out in the intervention manual, however, continue to adapt this as appropriate in the same way you have been doing.

- Month 1-2: bi-weekly
- Month 3-6: weekly
- Month 6-9: fortnightly
- Month 9-12: monthly

The length of the phone call may be much shorter depending on what is discussed.

The content of the phone call should be guided by the telephone coaching instructions below.

Some participants won't be suitable for telephone calls. If the participant is unable to engage with telephone coaching the carer should be contacted to determine if they may be able to use the telephone coaching to support the participant. If the telephone coaching is of no benefit to either the participant or the carer, then a courtesy telephone call should be given each month to keep in touch with the carer or participant as appropriate.

Final sessions should be carried out via the telephone as appropriate; these should be followed up with an end of therapy letter and any follow up material being provided using the post or email if appropriate.

For peer review only

New Intervention Group Participants

Intervention group participants seen by the research team but not yet seen by therapy team, or who are in the assessment phase of the intervention, should be informed that they are not going to receive the PrAISED intervention until the current restrictions are lifted.

Control Group Participants

If you have completed the first control visit you can carry out up to two follow up visits by telephone as per the guidance below. If the first control visit has not yet been completed, please inform the participant that they are not going to receive the PrAISED intervention until the current restrictions are lifted.

Therapy Visit Log

Continue to complete the therapy visit log, via the hyperlink, for all telephone calls. Please put telephone coaching in the comments box.

Medium-Term Plan

It is expected that PrAISED therapy staff at each site will deliver the immediate plan outlined above.

However, as the situation changes a medium-term plan (outlined below) may come into action.

If sites cannot deliver the telephone coaching sessions due to therapy staffing difficulties, the university staff may have capacity to be able to support. The PI from each site must contact the University as soon as possible if this happens. For university staff to be able to do the telephone coaching sessions effectively, we will need to know:

- the participant's details (e.g., contact telephone number for them and the carer/informant)
- a synopsis of the previous intervention session and what they are currently working on

As each site is using different participant documentation systems, the PIs should liaise with Sarah Goldberg or Rebecca O'Brien, to form a contingency plan on how this will happen and how information is to be transferred and stored.

Telephone Coaching Instructions

Before making the telephone call make sure you have looked at NHS England current advice for the client group you are dealing with, as this is likely to change on a regular basis (https://www.nhs.uk/conditions/coronavirus-covid-19/). Participants may have concerns about their current situation that need answering before the participant will engage in coaching.

- Explain who you are and why you're calling.

For peer review only

- Ask how they are and discuss any immediate concerns (they may need signposting as appropriate).
- Review their current activity and exercise plan.
- Review what they are currently doing during their day.
- Be aware that for many participants all their activities may have stopped.
- Form a plan of what they can do within the **current** restrictions. For example, currently people are advised it is ok to walk outside as long as they stay 2m away from other people.
- Help them to make a daily plan of activities. For example, doing exercises more frequently, or if they are no longer walking outside can they walk in the garden or up and down the stairs to get some cardiovascular exercise.
- Advise against sitting for long periods of time. For example, use a timer to remind yourself to get up or get up during advert breaks in television programmes.
- If the person is able to and wants to, they could put you on speaker phone while you go through their exercise programme with them. Only do this if they have the capacity to do this with their telephone. This could also be done with their carer or family member or named informant.
- Be aware people may be feeling quite worried and/or low in mood. You may need to discuss the benefits of, and encourage them to continue to carry out daily activities or routines, such as getting dressed, or taking meals on time.
- Participants may raise safeguarding issues such as identifying they are low on medication and there is no one to help them with this. This will need to addressed using the usual safeguarding procedures.
- If participants are complaining of COVID 19 symptoms they should be encouraged to follow the current advice from NHS direct or to phone 111.

It is expected these telephone coaching guidelines will evolve as PrAISED therapists start conducting these sessions. Guidance can come from outside sources, e.g., RCOT have recently shared this online https://www.rcot.co.uk/staying-well-when-social-distancing. It is important that we share good practice and suggestions and will discuss these guidelines during our PrAISED Therapist Teleconferences.

For peer review only

Appendix 2. Template for analysis of audio-recordings of therapists' remote sessions

Principle	Description	Examples	Rater 1*	Rater 2*
**Intensive**	Physical activity must be performed for at least 150 minutes per week. Participants may require more or less intensive support to achieve this.	Does therapist ask about activity times or amount of activity done over the last week? Do they discuss and agree with participant level of intensity of support required and frequency of next visits? Do they discuss activity plans for the upcoming week?		
**Tailored**	The therapist must work with participant to select and tailor physical exercise / activities that will be of most benefit and interest	Does the therapist make the participant feel they are in control of the activities to be done? For example do they ask whether the participant wants to do the activity? Does the participant seem to enjoy doing it? Is the participant given choices around exercise/activity? Does the therapist make recommendations on activity/exercise based on what the participant has said, or what they have observed the participant do?		
**Challenging**	The tasks must be challenging	Are the tasks challenging enough for participant, but still within their capabilities (i.e. realistically achievable)?		
**Progressive**	The tasks must be progressive	Is the therapist increasing the challenge of the task progressively (even within the same session)? Do they discuss progressing the tasks, now or in the future?		
**Promoting / improving independence**	The tasks must promote or improve independence (ability to complete tasks without dependence on others)	Is the therapist asking the participant to carry out activities independently or working towards them being independent? (e.g. personal, domestic or leisure ADLs, navigating the kitchen, making tea). Do they		

For peer review only

		discuss how the participant could be more independent or set goals for them to do activities independently?		
**Supporting in ADLs or exercise**	The therapist must work with participants to find ways in which the participant can do daily tasks and activities	Does the therapist discuss strategies (e.g. photos, instructions, carer input) with participant to enable them to do their ADLs or exercise? Do they explain how to do them? Do they use a clear language and practical example to support them?		
**Supporting dual-tasking**	The therapist must challenge the participant to complete two exercises at once	Does the therapist ask the participant to do tasks where the mind and the body work at the same time (e.g. walking and counting)? This could be either with the exercise programme or through a functional activity.		
**Accessing the environment**	The therapist must consider ways to maximise physical activity and exercise in the participant's home	Does the therapist ask about, advises on, suggests or gives information on activities that can be done inside the home? Does the therapist discuss full access of the person's property?		
**Embracing positive risk-taking**	Tasks must encourage positive risk-taking and only be discouraged if safety could be compromised	Does the therapist encourage the participant to do tasks where there is a degree of calculated risk? Does the therapist expose the participant to unnecessary risk of harm? Does the therapist discuss positives and negatives of doing more risky activities? Does the therapist use the risk enablement paperwork? Does the therapist consider risk management strategies or contingency plans, when discussing more risky activities?		
**Using Self-Determination Theory principles**	Contact must respond to the human needs for competence (feeling capable of doing the tasks), autonomy (being	Does the therapist give unconditional support and encouragement to boost the participant's confidence? Does the therapist empower the		

For peer review only

	in control of the programme and the tasks)** and relatedness (feeling cared for and connected to the therapist)	participant, by giving them control over the tasks and other aspects of the programme? Is the therapist relatable and working actively to build a human connection with the participant? Does the therapist use the 12 motivational strategies provided by PrAISED team?		
**Assisting in habit formation**	Therapist must assist the participant to develop a habit of being physically active	Does the therapist find ways to integrate the activities into the participant's routine? Do they check that the participant is forming a habit of doing physical activity? Does the therapist use the habit forming strategies provided by PrAISED team?		
**Using tapering to promote self-management**	Therapist must grade the amount of support and supervision provided to participant, to make them more responsible of the activity as time progresses	Does the therapist discuss and agree with participant on the level of intensity of support required to do the tasks and the frequency of next contacts? Is the therapist progressively reducing support (even within the session)? Does the therapist discuss reducing the level of support as programme progresses? Does the therapist use the tapering strategies provided by PrAISED team?		
**Promoting long-term engagement**	The therapist must support the participant to develop intrinsic motivation to ensure that they participants remain active over time	Does the participant seem to enjoy the activity plan? Does it seem that the participants might be able / willing to keep doing the activities over time? Do the therapist work to ensure this, by exploring participant's views?		
**Goal Setting**	The therapist must set goals with the participant that are specific to their interests, functional and active	Does the therapist discuss goal setting with the participant? (sets new goals, review existing goals, adapt/change goals)		

For peer review only

	Does the goal or action plan associated with it lead to the participant doing regular active tasks?		

* Rate as: 1=Visit following principle; 2=visit not following principle; 0=Principle not applicable

For peer review only
